# Functional characterization of the biogenic amine transporters on human macrophages

**DOI:** 10.1172/jci.insight.151892

**Published:** 2022-02-22

**Authors:** Phillip M. Mackie, Adithya Gopinath, Dominic M. Montas, Alyssa Nielsen, Aidan Smith, Rachel A. Nolan, Kaitlyn Runner, Stephanie M. Matt, John McNamee, Joshua E. Riklan, Kengo Adachi, Andria Doty, Adolfo Ramirez-Zamora, Long Yan, Peter J. Gaskill, Wolfgang J. Streit, Michael S. Okun, Habibeh Khoshbouei

**Affiliations:** 1 Department of Neuroscience, University of Florida College of Medicine, Gainesville, Florida, USA.; 2 Department of Pharmacology and Physiology, Drexel University College of Medicine, Philadelphia, Pennsylvania, USA.; 3 Neuronal Signal Transduction Group, Max Planck Florida Institute for Neuroscience, Jupiter, Florida, USA.; 4 Interdiscipinary Center for Biotechnology Research, University of Florida, Gainesville, Florida, USA.; 5 Department of Neurology,; 6 Norman Fixel Institute for Neurological Disease, Department of Neurology, and; 7 Center for Translational Research in Neurodegenerative Disease, University of Florida College of Medicine, Gainesville, Florida, USA.

**Keywords:** Inflammation, Neuroscience, Innate immunity, Macrophages, Transport

## Abstract

Monocyte-derived macrophages (MDMs) are key players in tissue homeostasis and diseases regulated by a variety of signaling molecules. Recent literature has highlighted the ability for biogenic amines to regulate macrophage functions, but the mechanisms governing biogenic amine signaling in and around immune cells remain nebulous. In the CNS, biogenic amine transporters are regarded as the master regulators of neurotransmitter signaling. While we and others have shown that macrophages express these transporters, relatively little is known of their function in these cells. To address these knowledge gaps, we investigated the function of norepinephrine transporter (NET) and dopamine transporter (DAT) on human MDMs. We found that both NET and DAT are present and can uptake substrate from the extracellular space at baseline. Not only was DAT expressed in cultured MDMs, but it was also detected in a subset of intestinal macrophages in situ. Surprisingly, we discovered a NET-independent, DAT-mediated immunomodulatory mechanism in response to LPS. LPS induced reverse transport of dopamine through DAT, engaging an autocrine/paracrine signaling loop that regulated the macrophage response. Removing this signaling loop enhanced the proinflammatory response to LPS. Our data introduce a potential role for DAT in the regulation of innate immunity.

## Introduction

Monocytes and monocyte-derived macrophages (MDMs) are heterogenous populations that serve as critical components of the immune system. MDMs arise when monocytes engraft into tissues to replenish the resident macrophage pool, such as in the gut, dermis, heart, and lung (1) or in response to inflammatory signals (2). Once engrafted, MDMs become transcriptionally distinct from circulating monocytes adopting a unique phenotype depending on their tissue-specific microenvironmental niche (3). Fundamental MDM functions include phagocytosis, cytokine production, and antigen presentation, and defective or aberrant macrophage functions have been associated with inflammatory (4), autoimmune (5), and neurological diseases (6, 7).

Biogenic amines — such as norepinephrine, in particular — have been noted for their ability to dynamically regulate macrophage function (8). A subset of intestinal macrophages express β-adrenergic receptors that engage a tissue-protective phenotype (9), and adipose macrophages adjacent to sympathetic terminals express a functional norepinephrine transporter (NET) that modulates the proinflammatory state and thermogenesis (10), indicating that the biogenic amine transporter activity itself can influence the immune system.

Recently, dopamine has been reported to have its own immunomodulatory properties independent of norepinephrine (11, 12). Dopamine is found in the kidney, adrenal glands, carotid body, and gut in concentrations high enough to activate dopamine receptors (13). Dopamine receptor activation has a wide range of effects on macrophage functions, including phagocytosis, cytokine production, and inflammasome activation (14, 15). The variance in the results might be partially explained by the different concentrations of dopamine used (and, thus, the activated dopamine receptor type) and whether the study was done in primary immune cells from humans or mice, in immortalized cell lines, or in immune-like heterologous cells. The frequently conflicting results indicate that current knowledge of dopamine signaling in immune cells is incomplete.

In the CNS, dopamine signaling is a dynamic, tightly regulated process with the dopamine transporter (DAT) serving as the master regulator of dopamine transmission (16–19). DAT can regulate dopamine signaling by uptake of extracellular dopamine into the cell and efflux of intracellular dopamine out of the cell (20–22). Although macrophages express the DAT, how immune cells may regulate dopamine signaling via DAT, or how DAT activity itself may modulate macrophage phenotype, is largely unknown. Elucidating DAT function in human primary macrophages will help provide a more comprehensive view of dopamine’s role in immune function and could be essential for guiding future studies targeting the dopamine system as an immune modulator.

In this study, we aimed to first characterize the biology of biogenic amine transporters on primary human MDMs. Employing multiple complementary approaches such as flow cytometry, quantitative PCR (qPCR), immunoblotting, and fixed- and live-cell microscopy, we found that primary MDMs from healthy human subjects express a functional NET and DAT, but not serotonin transporter (SERT). Furthermore, we found that, in addition to MDMs in vitro, a subset of human intestinal macrophages express DAT in situ. While NET expression and its activity on human macrophages are known, the discovery of functional DAT on these cells was unexpected. Importantly, we discovered that DAT activity can modulate the macrophage response to endotoxin, independent of NET. We attributed this to LPS-induced DAT-mediated efflux of dopamine and enhancement of autocrine dopamine signaling introducing a potentially novel role for DAT as a potential immunomodulatory rheostat.

## Results

### Human monocytes and macrophages express NET and DAT, but not SERT.

**Figure 1:**
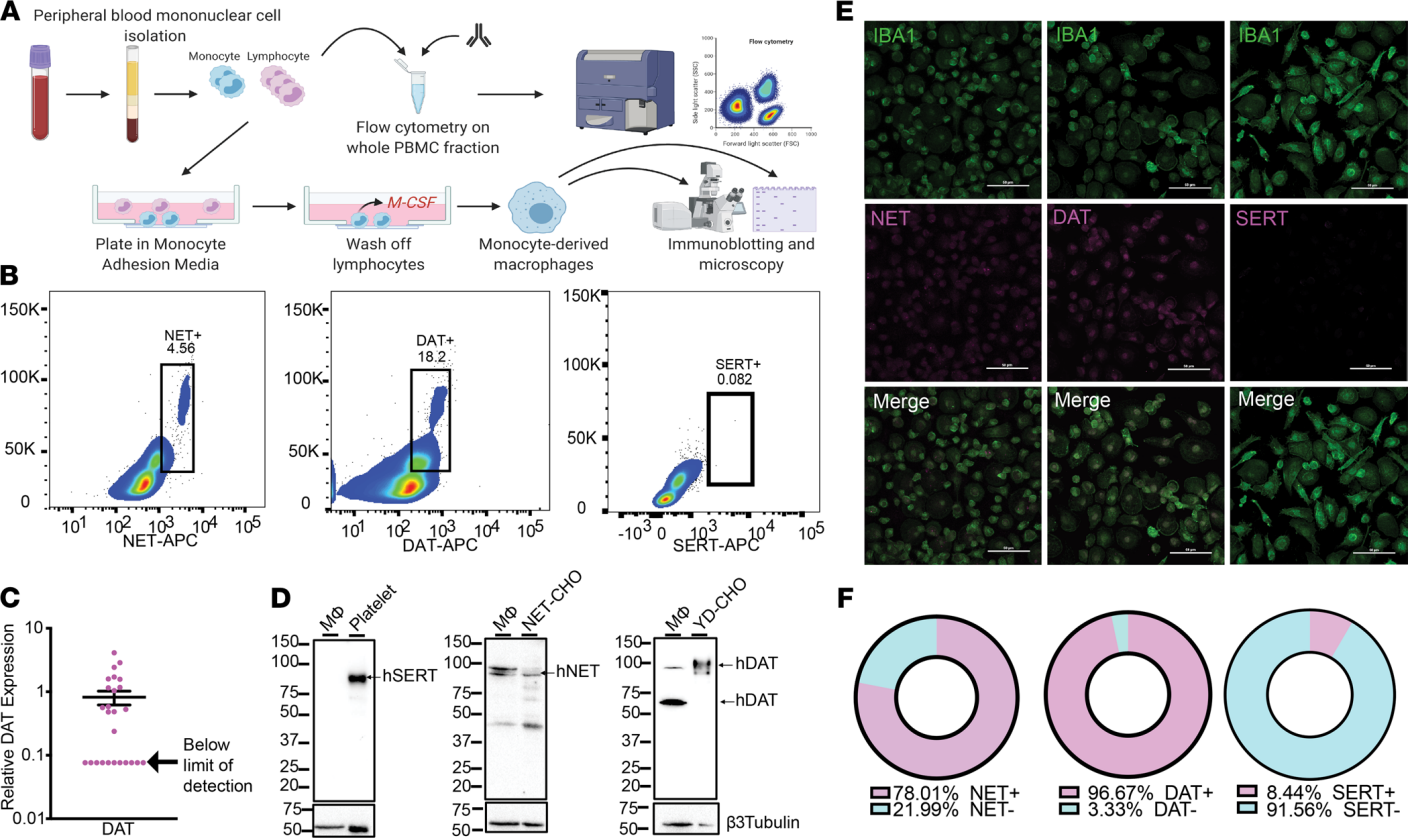
Human monocytes and monocyte-derived macrophages express NET and DAT, but not SERT. (**A**) Schematic depicting the isolation of PBMCs from human whole blood via density-dependent centrifugation with Ficoll. A fraction of the isolated PBMCs was used for flow cytometric analysis, and the remaining cells were plated in monocyte-adhesion media with autologous serum and allowed to differentiate into monocyte-derived macrophages. (**B**) Density plots of flow cytometry data on acutely isolated human PBMCs show that approximately 18.2% of circulating monocytes are DAT^+^ and approximately 4.56% of circulating monocytes are NET^+^ (scatter plots representative of 3 independent experiments). SERT was not detected on monocytes. (**C**) qPCR on cultured human monocyte-derived macrophages indicates that the mRNA for DAT protein is expressed in these cells (*n* = 26). (**D**–**F**) Cultured human monocyte-derived macrophages were either prepared for Western blot analysis (**D**), or immunocytochemistry (**E** and **F**). Representative Western blots from lysates of cultured human monocyte-derived macrophages probed for SERT, NET, or DAT. Human monocyte-derived macrophages did not express SERT (positive control: human platelets) but did express both NET (positive control: NET-expressing CHO cells) and DAT (positive control: YFP-DAT-expressing CHO cells) (*n* = 3 independent experiments). (**E**) Representative confocal images of human monocyte-derived macrophages immunostained for IBA1 and either NET, DAT, or SERT. IBA^+^ cells (macrophages) were positive for NET and DAT, but not SERT (*n* = 3 independent experiments). (**F**) Threshold-based quantification of NET^+^ (top), DAT^+^ (middle), and SERT^+^ (bottom) IBA1^+^ macrophages based on images in **E** indicating that 78% of macrophages were NET^+^, nearly 97% were DAT^+^, and only 8% were SERT^+^.

To study the biology of biogenic amine transporters on primary human monocytes and MDMs, we isolated blood mononuclear cells from peripheral blood (Figure 1A). Using a previously published protocol generated in our lab (23), we measured the percent of freshly isolated monocytes expressing either NET or DAT. Approximately 18% of monocytes were DAT^+^; whereas approximately 4%–5% of monocytes were NET^+^ and no monocytes were positive for SERT (Figure 1B; gating strategy shown in Supplemental Figure 1; supplemental material available online with this article; https://doi.org/10.1172/jci.insight.151892DS1).

Circulating monocytes undergo transcriptional reprogramming upon differentiation into MDMs (3). This prompted us to inquire whether biogenic amine transporter expression observed in circulating monocytes was retained in MDMs. To this end, we differentiated monocytes into MDMs in vitro over 6–7 days (24) (Figure 1A). qPCR of cultured human monocytes revealed that the mRNA for DAT was also expressed in these cells (Figure 1C), albeit in low amounts. Notably, the mRNA levels for some donors were below the limit of detection. Western blot analysis of MDM lysates confirmed that differentiated MDMs expressed DAT at the protein level (positive control, YFP-DAT-expressing CHO cells) in addition to NET (positive control, NET-expressing CHO cells), but not SERT (positive control, human platelets; Figure 1D). Our data are consistent with a recent study showing expression of NET in murine and human adipose-tissue macrophages associated with sympathetic nerve terminals (10), but that study did not probe for DAT. Hence, the finding of DAT expression on human MDMs is potentially novel. We validated these findings using immunofluorescence staining and confocal microscopy. Consistent with our Western blot data, Iba1^+^ cells showed signals for both NET and DAT, but not for SERT (Figure 1E). Approximately 97% of cultured MDMs were DAT^+^ (Figure 1F) compared with only 18% of circulating monocytes, indicating that the in vitro differentiation promoted a molecular reprogramming resulting in consistent DAT expression. Taken together, the data support the interpretation that human MDMs express both NET and DAT, but not SERT. To account for the small percentage of SERT^+^ macrophages, unless otherwise noted, all bath solutions in experiments going forward contained a SERT inhibitor, fluoxetine.

### NET and DAT in human MDMs are membrane bound and functional.

**Figure 2:**
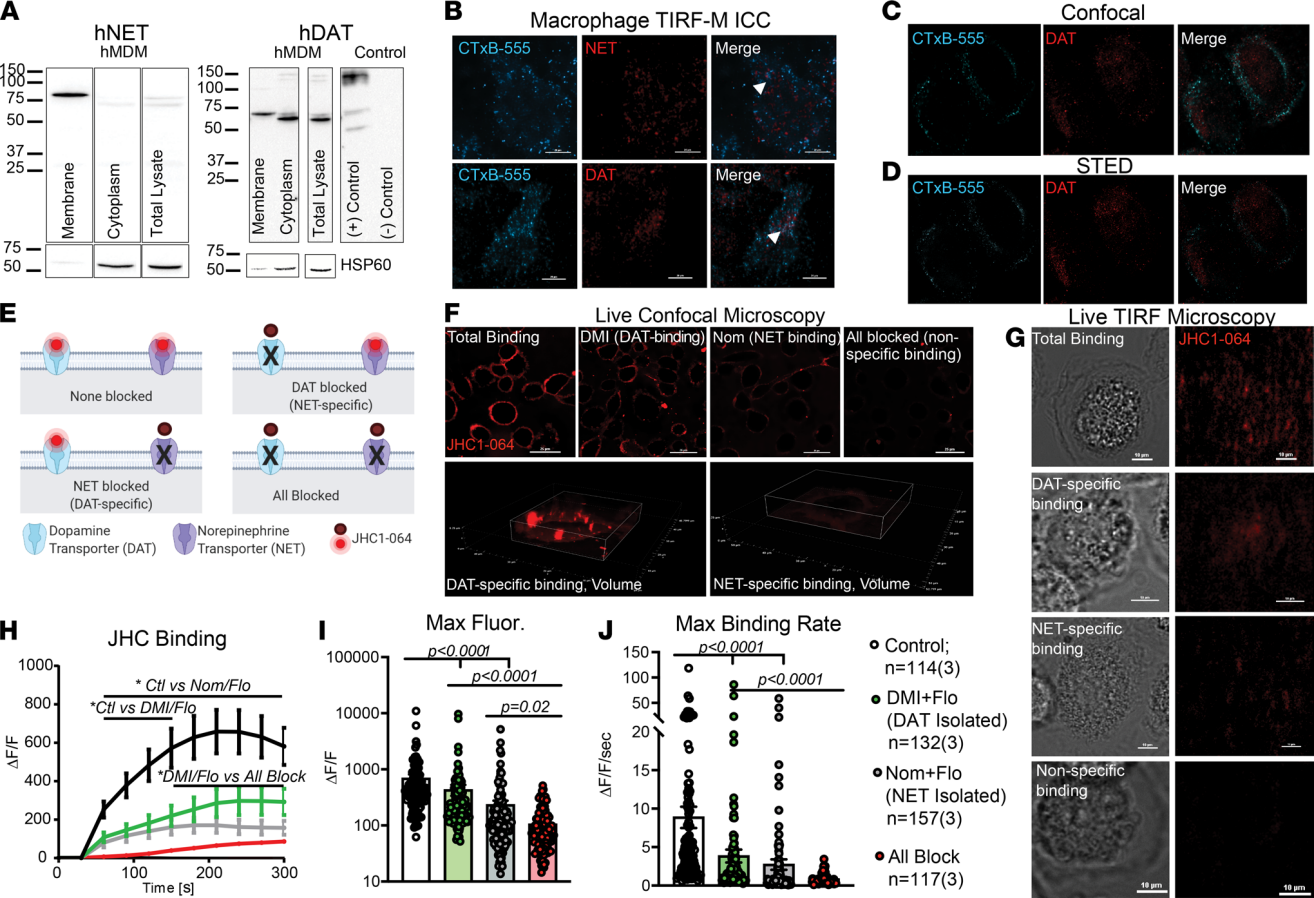
Human monocyte-derived macrophages express DAT and NET that are localized to the plasma membrane. (**A**) Representative immunoblots for NET and DAT from macrophage intracellular and membrane fractions separated via biotinylation assay (splice sites indicated by solid lines). (**B**) Representative 40× total internal reflective fluorescence microscopy (TIRF-M) images of cultured macrophages labeled with CTxB-555 and either NET or DAT antibodies. CTxB-555 punctae appeared on the basal membrane, along with scattered punctae of NET (top) and DAT (bottom). Some punctae of CTxB colocalized with NET or DAT (arrowheads, *n* = 4 experiments). (**C** and **D**) Representative confocal images (**C**, *n* = 4 experiments) or stimulated emission depletion (STED) images (**D**, *n* = 2 experiments) on cultured human macrophages labeled with CTxB-555 and for DAT with some of the DAT signal colocalizing with the CTxB-555 signal at or near the membrane. (**E**) Schematic of the experimental design for JHC1-064 binding assay to identify NET-specific and DAT-specific binding. (**F** and **G**) Live-cell confocal (60×) and TIRF-M (40×) images of JHC1-064 binding to macrophages in conditions shown in **E**. (**H**) Quantifying the JHC1-064 signal from **F** shows that blocking NET or DAT decreased JHC1-064 binding. Blocking both transporters further decreased the JHC1-064 signal on macrophages (2-way ANOVA with Tukey’s test for multiple comparisons, *P* < 0.05). (**I** and **J**) The magnitude (**I**) and rate (**J**) of JHC1-064 binding to human macrophages were decreased by the addition of Nom or DMI. (**I**) Control versus DAT-specific (*P* < 0.0001), control versus NET-specific (*P* < 0.0001). (**J**) Control versus DAT-specific (*P* = 0.0004), control versus NET-specific (*P* < 0.0001). Blocking all 3 transporters further decreased the magnitude (**I**, *P* < 0.0001) and rate (**J**, *P* < 0.0001) of JHC1-064 binding. Images and data in **F**–**J** are from *n* = 114–157 cells/group from 3 independent experiments. Statistical analysis in **I** and **J** performed by Kruskal-Wallis test with Dunn’s test for multiple comparisons.

We next sought to determine whether DAT or NET are functional in human macrophages. To do so, we first investigated the subcellular distribution of NET and DAT in cultured MDMs (24). Using cell surface biotinylation assays followed by immunoblotting, both NET and DAT were detected in the biotinylated membrane fraction of MDMs at the appropriate molecular weights (Figure 2A). Notably, macrophages harbored an intracellular pool of DAT but not NET. To complement these data, we performed total internal reflective fluorescence microscopy (TIRF-M). We validated our TIRF-M in CFP-DAT-expressing CHO cells (Supplemental Figure 2A). Cultured MDMs were colabeled with Alexa Fluor 555–conjugated CTxB (CTxB-555) and with antibodies against NET or DAT. TIRF-M readily identified the plasma membrane marked CTxB-555 labeling (25, 26). In concordance with our biotinylation data, scattered NET and DAT punctae were also detected in the TIRF plane, some of which colocalized with CTxB-555 punctae (Figure 2B). To confirm the TIRF-M data, we employed stimulated emission depletion (STED) super-resolution microscopy and confocal microscopy on macrophages colabeled with CTxB-555 and anti-DAT antibody (with an Alexa Fluor 647 secondary antibody). Both confocal (Figure 2C) and STED (Figure 2D) microscopy showed modest colocalization between CTxB-555 and DAT, consistent with the abundant expression of GM1 at the plasma membrane and DAT localization to both the plasma membrane and intracellular compartment.

Due to the limited sample size available for STED microscopy experiments, we sought to further validate these findings by the use of JHC1-064, a membrane-impermeable fluorescent analogue of cocaine, which only fluoresces upon binding to outward-facing biogenic amine transporters at the membrane (27). First, we validated our ability to measure JHC1-064–DAT binding in CHO cells transfected with CFP-DAT as a positive control. CFP is excited by the 405 nm laser line and detected at 485 nm, whereas JHC1-064 excitation and emission are 561 nm and 617 nm, respectively; therefore, there is a minimal bleed-through between the 2 channels. Live cell confocal and TIRF-M experiments in CFP-DAT-expressing cells showed overlay between the CFP-DAT signal and the JHC1-064 signal (Supplemental Figure 2, B and C), demonstrating that JHC1-064 binds to DAT in these cells. The JHC1-064–DAT binding was blocked when the cells were pretreated with a DAT antagonist, nomifensine (Nom, 10 μM).

The DAT–JHC1-064 binding data in the CHO-DAT cells with or without Nom were used as positive and negative control groups to measure JHC1-064 binding to human macrophages. Live-cell microscopy required us to study NET and DAT activity separately. Sorting by FACS of live monocytes that were either NET^+^ or DAT^+^ (i.e., not double positive) was not possible, since these antibodies bind intracellular epitopes and would require permeabilization. Therefore, to isolate NET and DAT activity, we studied DAT or NET function in the presence of NET antagonists to isolate DAT-specific activity and vice versa (Figure 2E). Using live-cell confocal (Figure 2F) and TIRF-M (Figure 2G), we measured JHC1-064 binding in human macrophages in 4 different conditions: (a) no antagonists present, control condition representing total binding; (b) pretreatment with Nom (10 μM) to isolate NET/JHC1-064 binding; (c) pretreatment with desipramine (DMI, 10 μM) to isolate DAT/JHC1-064 binding; and (d) pretreatment with DMI and Nom, which blocks all specific binding. All conditions except for the “total binding” condition also contained fluoxetine (1 μM). As predicted, the highest JHC1-064 binding was detected when no antagonists were present. The magnitude of JHC1-064 binding (Figure 2, H and I; *n* = 114–157 cells from 3 experiments/group) was attenuated when macrophages were pretreated with DMI (*P* < 0.0001) or Nom (*P* < 0.0001), and it was further attenuated when all 3 antagonists were present (DAT-specific versus all block, *P* < 0.0001; NET-specific versus all block, *P* = 0.02). A similar pattern was observed in the rate of JHC1-064 binding (Figure 2J), although not all differences reached significance. These same trends for NET–JHC1-064 binding and DAT–JHC1-064 binding were qualitatively observed in TIRF-M (Figure 2G). While the detection of NET activity on human macrophages was anticipated, identifying the Nom-sensitive DAT activity on these cells was unexpected.

**Figure 3:**
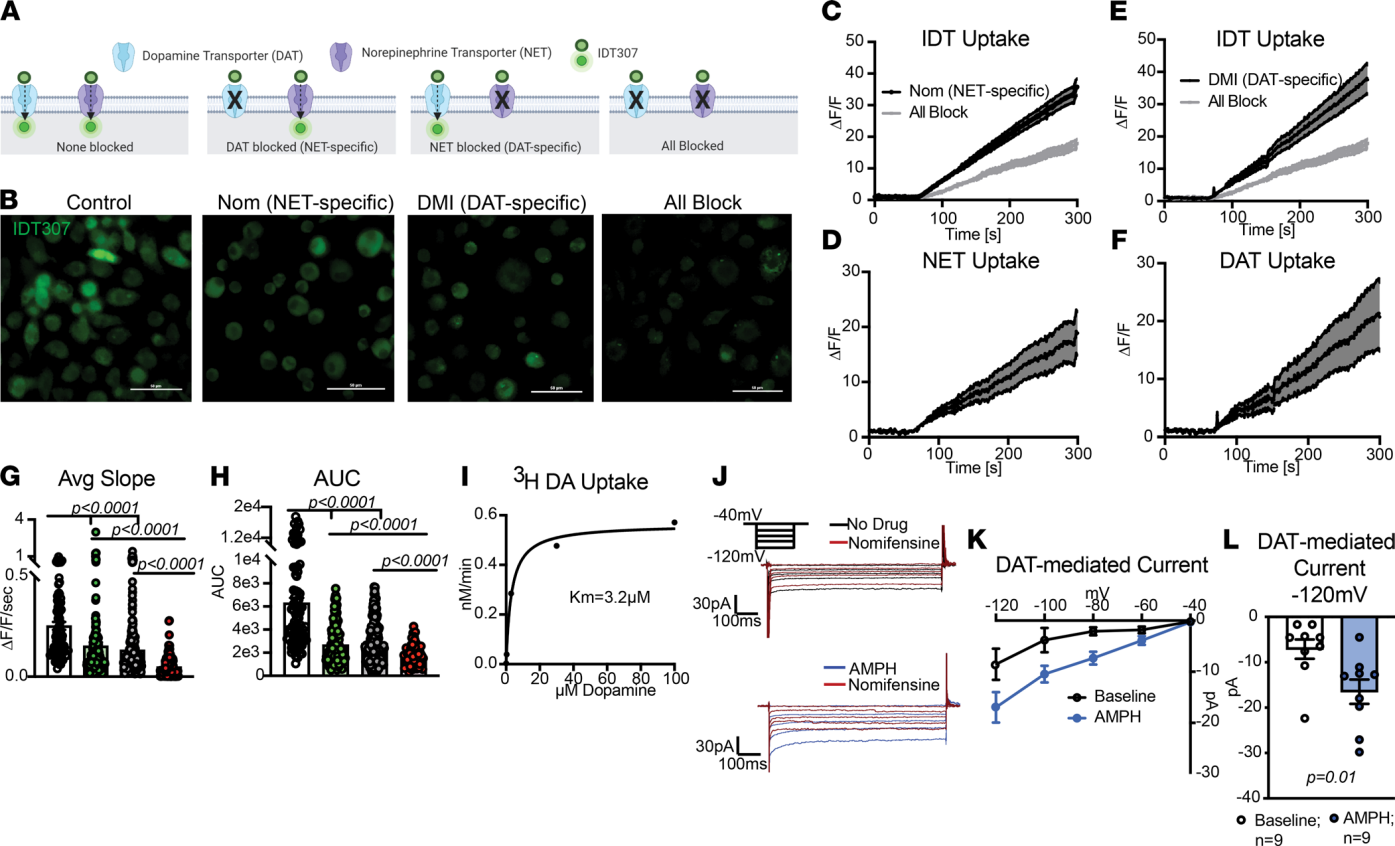
NET and DAT on human macrophages can work in uptake mode. (**A**) Schematic of the experimental design employing various conditions to measure DAT- versus NET-specific IDT307 uptake. (**B**) Representative 40× images of human macrophages following perfusion with IDT307 under conditions in **A**. (**C** and **E**). Quantification of IDT307 uptake as fold change in fluorescence in the presence of nomifensine (**C**), desipramine (**E**), or both antagonists (gray trace). (**D** and **F**) The nonspecific values (gray trace) were subtracted to show fold increase in NET- or DAT-mediated IDT307 uptake. (**G** and **H**) Blockade of either NET or DAT significantly decreased IDT307 uptake (average slope: control versus DAT-specific [*P* < 0.0001], control versus NET-specific [*P* < 0.0001]; AUC: control versus DAT-specific [*P* < 0.0001], control versus NET-specific [*P* < 0.0001]), and the multiantagonist cocktail further decreased IDT uptake (average slope: all block versus DAT-specific [*P* < 0.0001], all block versus NET-isolated; AUC: all block versus DAT-isolated, all block versus NET-specific [*P* = 0.0016]). Images and data in **B**–**H** are from *n* = 88–294 cells/group from 3 independent experiments. Statistical analysis in **G** was by Kruskal-Wallis test with Dunn’s test for multiple comparisons. Statistical analysis in **H** was by Brown-Forsythe and Welch ANOVA with Dunnett’s T3 multiple comparison’s test. (**I**) Nomifensine-sensitive uptake of tritiated dopamine (^3^H-DA) by cultured human macrophages shown as nM/min with a K_M_ of approximately 3.2 μM. Data from 4 experiments from 2 donors. (**J**) Representative traces of inward currents on human macrophages measured via whole-cell voltage-clamp with vehicle, after amphetamine (AMPH), and after nomifensine, a DAT antagonist. The bath solution contained NET and SERT antagonists. (**K**) The DAT-mediated inward currents were calculated by subtracting the nomifensine current from the current in the no drug recording (baseline) or amphetamine recording (AMPH). (**L**) Bar graph compares basal and amphetamine-induced DAT-mediated inward current at –120mV (right, *P* = 0.01, Mann-Whitney *U* test). Data are from 9 experiments/group.

Both NET and DAT canonically work to uptake extracellular substrate into the intracellular space. Therefore, we asked if NET and DAT in cultured human macrophages were capable of uptake. To this end, we used IDT307, a substrate for DAT and NET that fluoresces upon entry into the cell (28) (Figure 3A). We confirmed our ability to measure DAT-specific uptake with IDT in CFP-DAT-expressing CHO cells in the presence of vehicle (positive control) or Nom (10 μM, negative control; Supplemental Figure 3, A–C; *P* < 0.0001; *n* = 19–27 cells/group from 3 experiments). We repeated this assay in macrophages treated with the same 4 conditions described above: total binding (no antagonists), pharmacological isolation of NET activity (Nom), pharmacological isolation of DAT activity (DMI), and isolation of nonspecific activity (all block) (Figure 3B and Supplemental Video 1; *n* = 88–294 cells/group from 3 experiments). As above, all conditions except for “total binding” contained fluoxetine. Subtracting fluorescence of the nonspecific condition from either the NET-specific or DAT-specific conditions revealed notable and separate NET-mediated (Figure 3, C and D) and DAT-mediated (Figure 3, E and F) uptake capacities of human macrophages, respectively. Similar to the JHC1-064 assay, we observed maximum IDT307 uptake without any antagonists. The magnitude and rate of IDT307 uptake was attenuated by both the Nom (AUC, *P* < 0.0001; average slope, *P* < 0.0001) and DMI (AUC, *P* < 0.0001; average slope, *P* < 0.0001) conditions. Addition of all antagonists further attenuated uptake (Figure 3C; AUC, DAT versus all block, *P* < 0.0001; AUC, NET versus all block, *P* < 0.0001) (Figure 3, G and H; average slope and area under the curve DAT versus all block, *P* < 0.0001; average slope NET versus all block, *P* < 0.0001). These data indicate that both NET and DAT on human MDMs can uptake substrate from the extracellular milieu.

While NET-mediated uptake in adipose-resident macrophages has been shown (10), the finding of a separate DAT-mediated uptake was surprising. Therefore, we employed 2 additional complementary approaches to validate our findings. First, we used the classical tritiated dopamine uptake assay and found that human MDMs exhibited Michaelis-Menten-like kinetics for dopamine uptake, with a K_M_ of approximately 3.2 μM (Figure 3I). This is similar to the K_M_ observed in other DAT- expressing systems (29). Further confirming our findings human MDM showed a Nom-sensitive, DAT-mediated inward current similar to but smaller than positive controls (Supplemental Figure 3D). The Nom-sensitive current was increased following DAT activation with amphetamine (Figure 3, J–L; *P* = 0.0144). Altogether, our data suggest that both NET and DAT are expressed at the membrane of primary human MDMs and function in their canonical uptake mode in these cells. Next, we investigated whether DAT expression is limited to circulating monocytes and MDMs or other whether tissue-resident macrophages also express DAT.

### A portion of human gut-resident macrophages are DAT^+^.

A recent study reported that human sympathetic-nerve-associated adipose macrophages express a functional NET (10), but that study did not probe for DAT. While our data indicate that human monocytes and MDMs in vitro express DAT, cultured macrophages lack the tissue-derived signaling factors and, thus, do not faithfully recapitulate the phenotype of any tissue-resident macrophage (30). Thus, before proceeding, we asked if any human tissue macrophage populations expressed DAT in situ. The gut is a rich source of dopamine (13), and its resident macrophage pool is partially maintained by MDMs (31). Therefore, we hypothesized that human gut-resident macrophages express DAT.

**Figure 4:**
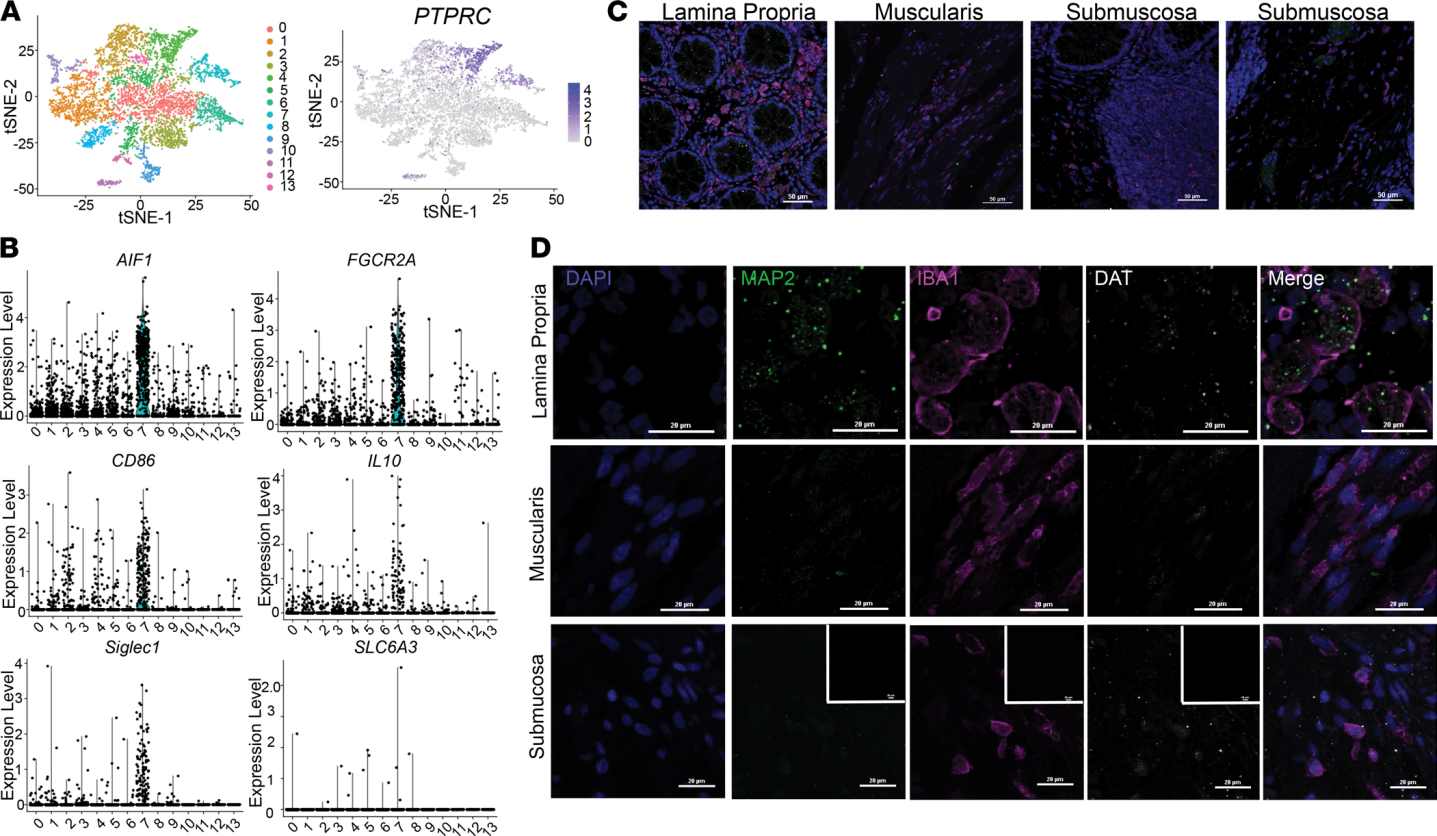
A subpopulation of human intestinal macrophages express DAT. (**A**) A previously published single-cell RNA-Seq data set was procured from NCBI’s GEO using the search terms “gut” and “macrophage”. The data set was analyzed using the R package Seurat to cluster the cells based off the 10 most significant principal components and dimensionally reduce the data using t-distributed stochastic neighbor embedding (t-SNE) plots yielding 13 different clusters of cells (left). Relative expression of PTPRC (CD45) was overlaid on the t-SNE plot (right). (**B**) Relative expression of macrophage markers AIF1/IBA1, FGCGRA, IL-10, CD86, and SIGLEC1 in addition to expression of SLC6A3 (DAT) in each of the 13 clusters represented as violin plots indicate that cluster 7 was enriched for macrophage markers and contained some SLC6A3-expressing cells. (**C**) Representative 40× confocal microscopy images of healthy human colon tissue labeled for IBA1 (macrophages), MAP2 (neurons), DAPI (nuclei), and DAT. Images were collected from various anatomical parts of the gut wall including the lamina propria (left), muscularis (middle-left), submucosa containing gut-associated lymphoid tissue (middle-right), and submucosa containing neuronal ganglia (right). All areas contained IBA1+ cells (macrophages). (**D**) High-magnification images from each of the anatomical regions shown in **C**. Lamina propria contained IBA1^+^ cells and IBA1^+^ cells enveloping MAP2^+^ areas. Some but not all submucosal IBA1^+^ cells were weakly DAT^+^, whereas muscularis macrophages were DAT^–^. Secondary-only negative controls shown as inset in the bottom of **D**. Images in **C** and **D** are from 3 independent experiments from 1 healthy human donor.

We curated a large single-cell RNA-Seq (scRNA-Seq) data set on biopsy samples of human colon from the NCBI Gene Expression Omnibus (GEO) (32). Clustering analysis of this data set using the Seurat pipeline as previously described (33) yielded 13 transcriptionally distinct clusters, which we annotated as various epithelial, muscle, and immune populations based on their differentially expressed genetic markers (Figure 4A). Searches for expression of the DAT gene (*SLC6A3*) showed low expression levels in several clusters — notably, cluster 7. Cluster 7 was also enriched in markers such as *AIF1*, *CD206*, *CD163*, *CYBB*, *CD86*, and *IL10*, consistent with the identity of tolerizing gut-resident macrophages (Figure 4B). Thus, these data suggest that, in addition to monocytes and cultured MDMs, the DAT is expressed in at least some human gut-resident macrophages.

An important limitation of the single-cell transcriptomic approach of a diverse sample population is the low sensitivity to low-abundance transcripts. To validate DAT protein expression in human gut-resident macrophages, we complemented the scRNA-Seq analysis with confocal microscopy in situ. In the gut, macrophage heterogeneity is partially governed by their anatomical niche within the intestinal wall (9, 34–36); therefore, we examined macrophage populations in the human colon lamina propria, submucosa, and muscularis. Tissues were immunolabeled for Iba1 (pan-macrophage marker), MAP2 (neuronal marker), and DAT and assessed using confocal microscopy (Figure 4C). Iba1^+^ cells were abundant in all locations. In the lamina propria, Iba1^+^ cells enwrapping MAP2^+^ puncta were DAT^+^, but it was difficult to determine whether the DAT signal was present on neurons or macrophages, or both (Figure 4D, top). However, in the submucosa, we found a subgroup of macrophages that were Iba1^+^DAT^+^ (Figure 4D, bottom, secondary-only negative control inset). Most of the Iba1^+^DAT^+^ cells were found near lymphoid-like follicles or MAP2^+^ ganglia, but they did not colocalize with MAP2^+^ areas, suggesting that this niche may be associated with DAT expression in gut macrophages. These data indicate that, in addition to circulating monocytes and cultured human MDMs, human gut-resident macrophages also express DAT in situ. It is important to note the limited sample size in this experiment. Next, we investigated whether DAT activity modulates macrophage immune functions such as cytokine secretion and phagocytosis.

### DAT activity modulates macrophage immune functions.

**Figure 5:**
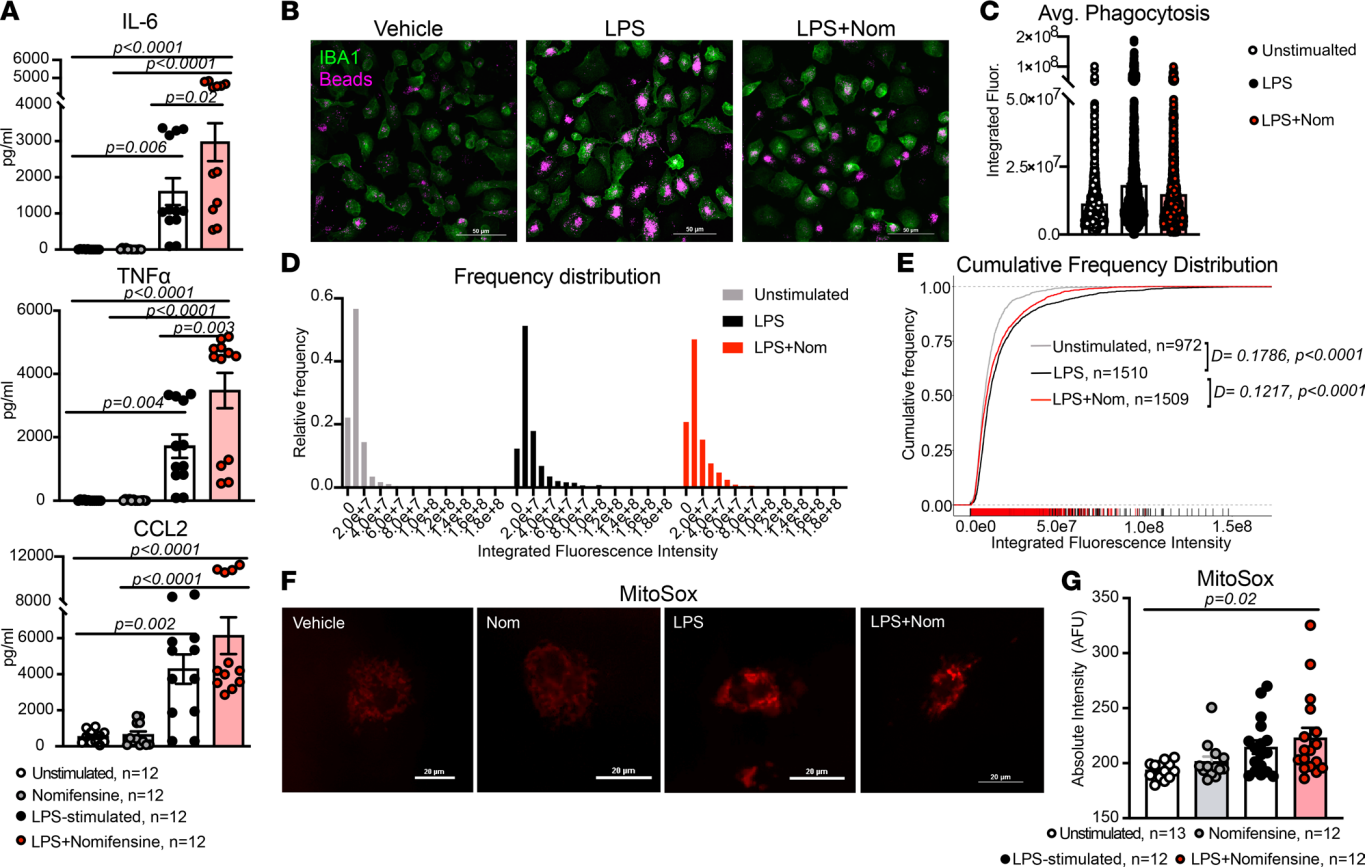
Inhibition of DAT enhances the proinflammatory program in response to LPS. (**A**) Cultured human macrophages were treated with vehicle, nomifensine, LPS, or LPS + nomifensine in the presence of NET/SERT blockade. LPS treatment significantly increased the secretion of all 3 soluble factors (IL-6, *P* = 0.02; TNF-α, *P* = 0.004; CCL2, *P* = 0.002). Nomifensine in the presence of LPS significantly increased the LPS-induced secretion of TNF-α (*P* = 0.003) and IL-6 (*P* = 0.02) and had a similar effect on CCL2 secretion. Data are from *n* = 12 experiments/group, and statistics were performed using a 1-way ANOVA with Tukey’s post hoc test. (**B**) Representative 40× images of cultured human macrophages treated with vehicle (media), LPS, or LPS + nomifensine and incubated with fluorescent latex beads, fixed, and labeled for IBA1. (**C**) The average integrated fluorescence intensity of phagocytic beads within macrophages showed a slight increase in LPS and a nonsignificant decrease with LPS + nomifensine. (**D**) Frequency histograms showing the skewed distribution of fluorescence intensity of phagocytic beads in macrophages across the 3 conditions. (**E**) Empiric cumulative frequency distribution curves of unstimulated, LPS-stimulated, and LPS + nomifensine–treated macrophages shows that LPS significantly increased phagocytosis compared with unstimulated condition (D-statistic = 0.1786, *P* < 0.0001). Cotreatment with LPS and nomifensine decreased macrophage phagocytosis back toward unstimulated levels (D-statistic = 0.1217, *P* < 0.0001, versus LPS-stimulated). Images and data in **B**–**E** are from *n* = 972–1510 cells/group across 3 independent experiments. Statistics were performed using Kolmogorov-Smirnov tests. (**F**) Representative images of cultured human macrophages treated as in **A** and incubated with MitoSox Red. (**G**) Only cotreatment with LPS + nomifensine produced a significant increase in mitochondrial superoxide levels compared with vehicle (*P* = 0.02). Images and data in (**F** and **G**) are from *n* = 12–13 experiments/group. Statistics were performed using a Kruskal-Wallis test with Dunn’s test for multiple comparisons.

Since our data indicate that human MDMs express a functional DAT (Figure 1, Figure 2, Figure 3, and Figure 4), we asked if DAT activity affected macrophage immune functions such as cytokine secretion and phagocytosis. We first investigated the effect of DAT inhibition on the cytokine profile of freshly isolated monocytes and MDMs. We treated freshly isolated monocytes (*n* = 6) and MDMs (*n* = 11–12/group) with either vehicle, Nom, LPS, or LPS + Nom for 24 hours. In MDMs, LPS significantly increased release of proinflammatory cytokines IL-6 (*P* = 0.006), TNF-α (*P* = 0.004), and CCL2 (*P* = 0.00 2). The effects of LPS on IL-6 and TNF-α were significantly increased in the presence of a DAT antagonist (Figure 5A; IL-6, *P* = 0.02; TNF-α, *P* = 0.003). In the absence of LPS, DAT blockade had no effect on the baseline release of these cytokines. We observed a similar effect in freshly isolated monocytes, with DAT blockade enhancing LPS-induced production of intracellular cytokines (Supplemental Figure 4). Notably, DAT inhibition had no effect on LPS-induced release of IL-1β (Supplemental Figure 5A), and NET inhibition had no significant effect on LPS-induced release of IL-6, TNF-α, or CCL2 (Supplemental Figure 5B). Thus, DAT activity may modulate the cytokine response independently of NET activity.

We next asked if DAT blockade had a similar effect on macrophage phagocytosis. Blocking NET, we utilized the experimental approach described by the Tsirka lab to quantify phagocytosis of fluorescent latex beads (Figure 5, B and C) via a publicly available pipeline for analysis (37). We detected a rightward shift in phagocytosis induced by LPS (D-statistic = 0.1786, *P* < 0.0001), consistent with an increase in phagocytosis capacity. Blockade of DAT in the presence of LPS resulted in a leftward shift, restoring the phagocytic capacity toward baseline levels (Figure 5, D and E; D-statistic = 0.1217, *P* < 0.0001). Importantly, the limitations of these experiments and the analyses are that the data only assess 2 macrophage functions and do not provide a comprehensive picture of the macrophage phenotype. Nevertheless, the increased proinflammatory cytokine release and decreased phagocytosis induced by DAT blockade suggest that loss of DAT activity skews macrophages toward a more proinflammatory state.

Mitochondrial health and mitochondrial oxidative stress are closely associated with macrophage phenotype and inflammatory function (38, 39). Therefore, we investigated whether DAT blockade during LPS stimulation affected mitochondrial oxidative stress. Macrophages were treated with MitoSox Red, a fluorescent reporter of mitochondrial superoxide species during LPS and Nom treatment (Figure 5F; all experimental conditions contained NET antagonist). Surprisingly, while LPS stimulation increased mitochondrial oxidative stress, this increase was not significant and only DAT blockade in the presence of LPS produced a significant elevation in mitochondrial superoxide levels (Figure 5G; *P* = 0.02). Although these data do not directly assess mitochondrial function, they support the notion that DAT blockade may affect mitochondrial health during inflammation via oxidative burden. Taken together, these data implicate DAT activity as a potential immunomodulator in response to LPS-induced immune stimulation.

### LPS decreases DAT-mediated uptake without changing DAT membrane levels.

To further examine the interaction between DAT activity and the macrophage inflammatory response, we examined the effects of LPS on DAT function, with the hypothesis that LPS alters DAT activity. Before investigating the potential LPS modulation of DAT activity, we first tested whether DAT on macrophages was subject to a well-characterized regulatory mechanism: PKC-induced internalization (40, 41). We monitored membrane DAT–JHC1-064 complexes in both macrophages and YFP-DAT-expressing cells before and after addition of phorbol myristate acetate (PMA, 1 μM), a PKC activator. Treatment with PMA decreased the TIRF-M footprint in both YFP-DAT cells (positive control group, *P* = 0.002) and macrophages (*P* = 0.002) (Supplemental Figure 6, A and B). In parallel experiments, we found that PMA also decreased the DAT-mediated uptake of IDT307 in human macrophages (Supplemental Figure 6, C and D). These data support the interpretation that DAT molecules on macrophages undergo PKC-induced internalization and that we can use microscopy to detect changes in surface DAT levels and activity on human macrophages.

**Figure 6:**
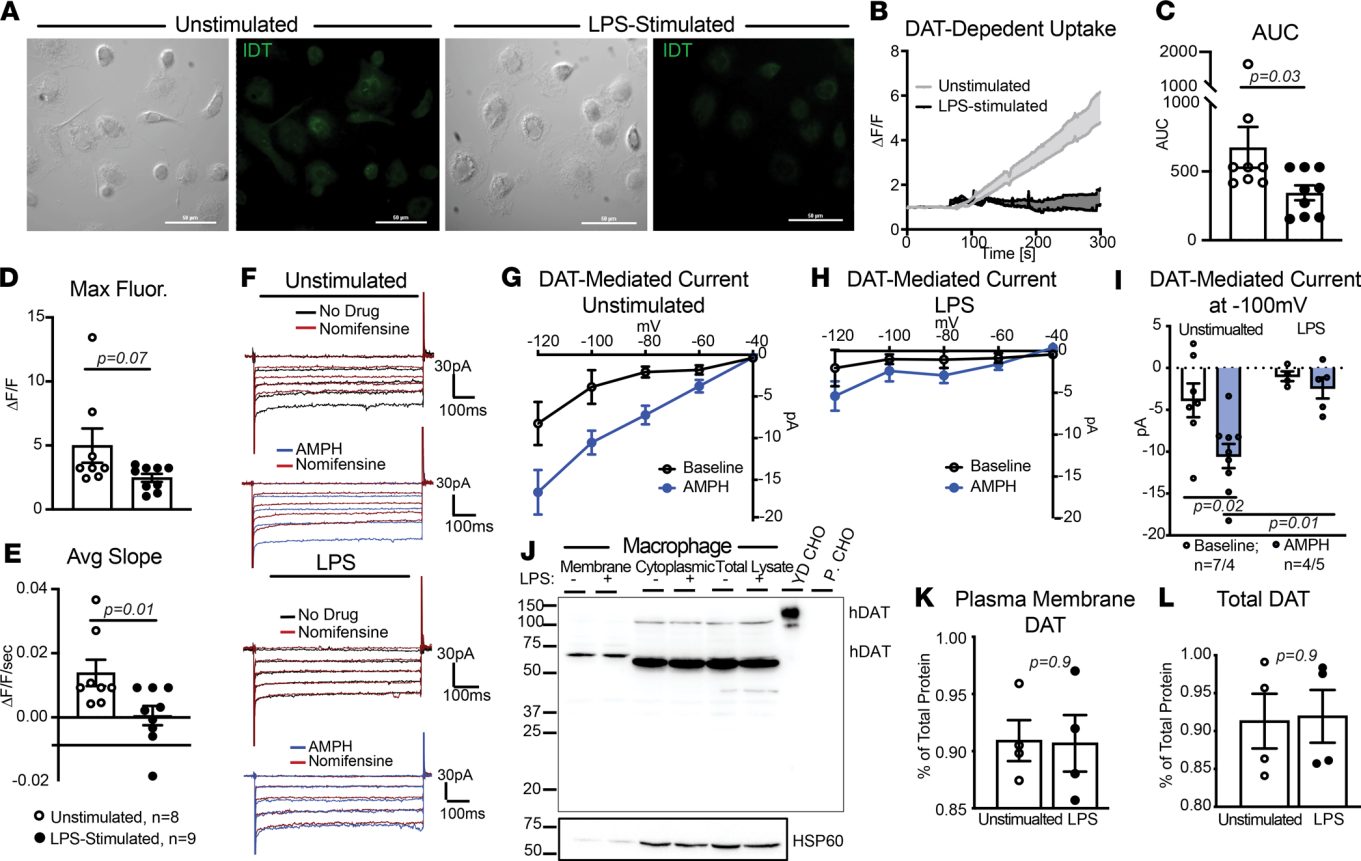
LPS stimulation decreases DAT-mediated uptake without affecting membrane or total DAT levels. (**A**) Representative 40× images of unstimulated or LPS-stimulated macrophages assayed for DAT-mediated IDT307 uptake. (**B**) DAT-mediated uptake, measured as fold increase in fluorescence intensity, was calculated as described in Figure 3F. Compared with unstimulated macrophages (control group), LPS stimulation decreased the DAT-mediated IDT307 uptake. (**C**–**E**) LPS-stimulation decreased the magnitude (**C**, AUC, *P* = 0.03, Mann Whitney *U* test), max fluorescence (**D**, *P* = 0.07, Mann Whitney *U* test), and the rate (**E**, average slope, *P* = 0.01, Mann Whitney *U* test) of DAT-dependent IDT307 uptake. Data are from *n* = 9 independent experiments/group. (**F**) Representative whole-cell current traces from unstimulated or LPS-stimulated macrophages. (**G** and **H**) The current–voltage curves show the DAT-mediated inward currents at different hyperpolarizing voltage steps for unstimulated (**G**) and LPS-stimulated (**H**) macrophages. DAT-mediated inward currents were calculated by subtracting the inward current in the presence of nomifensine from the inward currents at baseline or in the presence amphetamine (AMPH). (**I**) In unstimulated macrophages, amphetamine induced a nomifensine-sensitive inward current (*P* = 0.02); however, LPS stimulation decreased the amphetamine-induced DAT-mediated current (*P* = 0.01). Data in **F**–**I** are from 4–7 experiments/group, and statistical analysis was performed via 2-way ANOVA with Tukey’s post hoc test for multiple comparisons. (**J**) Representative immunoblot of membrane and cytoplasmic DAT in macrophages measured by surface biotinylation in the presence or absence of LPS. YFP-DAT-expressing CHO cells and parental CHO cells were used as positive and negative controls, respectively. Cytosolic and membrane fractions confirmed by HSP60 (lower panel). (**K** and **L**) Surface DAT (**K**) and total DAT levels (**L**) are expressed as percentage HSP60 ± SEM, *n* = 4 independent biological replicates. LPS did not alter membrane DAT levels (*P* = 0.9, unpaired 2-tailed *t* test).

We next shifted our focus to possible LPS-induced regulation of DAT activity. We repeated the IDT307 uptake assay in unstimulated macrophages and macrophages treated with LPS for 24 hours (Figure 6A). We observed a dramatic decrease in DAT-mediated IDT307 uptake in the LPS-stimulated macrophages (Figure 6, B–E; AUC, *P* = 0.03; max fluorescence, *P* = 0.07). We have shown that DAT-mediated uptake is also accompanied by a Nom-sensitive inward current in heterologous cells and on macrophages (Supplemental Figure 3D and Figure 3, J–L); therefore, to validate the LPS reduction of DAT-mediated IDT307 uptake, we performed patch-clamp recordings in whole-cell configuration in unstimulated and LPS-stimulated macrophages (Figure 6F). In unstimulated macrophages, we reproduced the earlier findings (Figure 3, J–L) and measured a Nom-sensitive, amphetamine-induced inward current that was significantly decreased by LPS stimulation (Figure 6, G–I; *P* = 0.02 and *P* = 0.01). These findings support the hypothesis that LPS stimulation decreases DAT-mediated uptake.

Decreases in DAT-mediated uptake and inward current can reflect decreases in membrane-localized DAT, decreases in total DAT, changes in outwardly versus inwardly conformational states of DAT, altered transport kinetics, or a combination thereof. Therefore, we investigated whether the reduced DAT-mediated uptake induced by LPS was due to decreased DAT membrane localization. Biotinylation of macrophages followed by immunoblotting showed that DAT was detected in both membrane and cytosolic fractions in both unstimulated and LPS-stimulated conditions (Figure 6J), and no difference was observed in either membrane DAT (*P* = 0.9) or total DAT (*P* = 0.9) between the experimental groups (Figure 6, K and L). These data suggest that LPS stimulation in human macrophages decreases DAT-mediated uptake without affecting total or surface DAT levels.

### LPS stimulation favors an efflux-promoting conformation of DAT.

**Figure 7:**
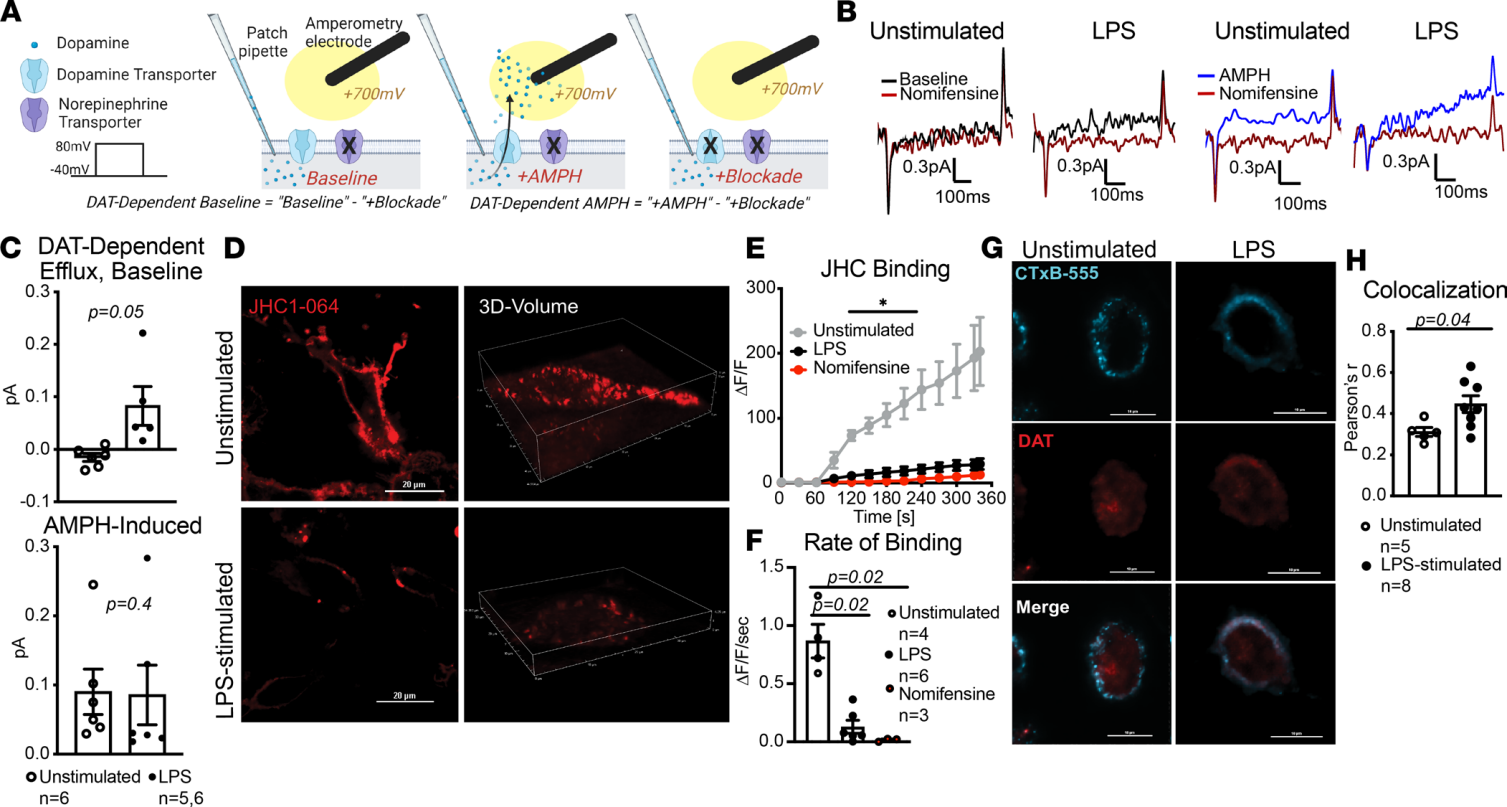
LPS-stimulation increased DAT-mediated dopamine efflux and decreased DAT–JHC1-064 binding. (**A**) Schematic representation of experimental design using simultaneous whole-cell patch-clamp and amperometry technique to measure DAT-mediated dopamine efflux. (**B**) Representative amperometric traces from unstimulated and LPS-stimulated human macrophages. (**C**) Bar graphs show basal or amphetamine-induced DAT-mediated dopamine efflux. While unstimulated macrophages did not exhibit measurable DAT-mediated dopamine efflux at baseline, LPS-stimulation significantly increased basal DAT-mediated dopamine efflux (top, *P* = 0.05, Welch’s 2-tailed *t* test) but did not further increase or decrease the amphetamine-induced dopamine efflux (bottom, *P* = 0.9, Mann Whitney *U* test). Data are from *n* = 5–6 experiments/group. (**D**) Representative 40× images of JHC1-064 binding to unstimulated and LPS-stimulated macrophages. (**E**) Quantification of fluorescence signal showed that LPS significantly decreased the magnitude of JHC1-064–DAT binding to levels similar to those seen with DAT blockade (nomifensine-treated, *P < 0.05, 2-way ANOVA with Tukey’s post hoc test for multiple comparisons). (**F**) The average rate of JHC1-064–DAT binding (average slope from **E**) was similarly decreased by LPS-stimulation (*P* = 0.02) and by presence of DAT-specific antagonist, nomifensine (*P* = 0.02). Data in **D**–**F** are from *n* = 3–6 independent experiments/group, and statistical analysis was performed by Brown-Forsythe ANOVA with Tukey’s post hoc test for multiple comparisons. (**G**) Representative images of unstimulated and LPS-stimulated macrophages labeled with CTxB-555 and DAT. (**H**) Quantifying colocalization between GM-1 and DAT using Pearson’s correlation coefficient showed LPS-stimulated macrophages has significantly increased DAT-CTxB colocalization compared with unstimulated macrophages (*P* = 0.04, unpaired 2-tailed *t* test). Images and data in **G** and **H** are from *n* = 5 and 8 experiments for unstimulated and LPS-stimulated groups, respectively.

We and others have previously shown that, in addition to uptake, DAT can engage in reverse transport, termed efflux, which is a process by which it transports the substrate into the extracellular space (20, 42, 43). DAT-mediated dopamine efflux is associated with drugs abuse (44–46), ADHD (17, 47), and autism (18, 48). Since LPS decreased DAT-mediated uptake without affecting membrane DAT levels, we hypothesized that LPS might alter DAT activity to favor an efflux promoting conformation. We tested this hypothesis using simultaneous patch-clamping and amperometry (20, 49, 50) (Figure 7A) to measure the DAT-dependent dopamine efflux in both unstimulated and LPS-stimulated macrophages (Figure 7B). To measure the DAT-mediated dopamine efflux, NET and SERT antagonists were present in the bath solution. We did not detect dopamine efflux at baseline in unstimulated macrophages; however, LPS-stimulated macrophages exhibited a significantly higher DAT-dependent dopamine efflux at baseline (Figure 7C, top; *P* = 0.05). Notably, amphetamine did not further increase or decrease the LPS-induced DAT-mediated dopamine efflux (Figure 7C, bottom; *P* = 0.9). These data collectively support the idea that LPS promotes DAT-mediated dopamine efflux in macrophages. To our knowledge, this is the first report of a pathophysiological case of DAT-dependent dopamine efflux, as this function has traditionally been studied in cases of genetic mutations (17, 18, 47) or exogenous drugs (20, 44, 45).

DAT-dependent dopamine efflux is associated with an inwardly facing conformation, whereas uptake is associated with an outwardly facing conformation. This prompted us to investigate if LPS induced an inward facing conformation of DAT. To assess the levels of inward- versus outward- facing conformation of DAT, we used JHC1-064, as this compound is membrane impermeable and binds to the outwardly facing conformation of biogenic amine transporters (51) (Figure 2E). Similar to pervious experiments, the bath solution contained NET and SERT antagonists. We used confocal microscopy to measure JHC1-064–DAT binding in unstimulated and LPS-stimulated macrophages (Figure 7D). LPS stimulation significantly reduced the magnitude (Figure 7E; *P* < 0.05) and rate (Figure 7F; *P* < 0.0001) of JHC1-064–DAT binding. Since LPS stimulation did not reduce surface DAT levels (Figure 6, J–L), the decreased JHC1-064–DAT binding observed in LPS-stimulated macrophages suggests that there were fewer outward-facing DAT molecules at the membrane, supporting the notion that LPS may promote an inward-facing, efflux-promoting DAT conformation on human macrophages.

The inward-facing conformation of DAT has been reported to localize to GM1 (52, 53) and syntaxin 1a–enriched (50) microdomains of the plasma membrane. Comparing the colocalization of DAT and GM1 marker CTxB-555 using fixed-cell confocal microscopy (Figure 7G) revealed LPS stimulation significantly increased DAT–CTxB-555 colocalization (Figure 7H; *P* = 0.04). This suggests that, following LPS stimulation, a larger proportion of DAT is distributed or stabilized in the GM1-enriched domains of the plasma membrane. It is important to acknowledge the dynamic nature of DAT activity — it is not an uptake-or-efflux dichotomy. Instead, there is a balance between DAT localizations and activities that summate to give a bulk picture. In this context, our findings support the interpretation that LPS stimulation favors an inward-facing and efflux-promoting DAT conformation that is potentially distributed in the GM1-enriched regions of the membrane on human macrophages.

### CD14-dependent regulation of DAT activity engages an autocrine loop to modulate macrophage immune response.

**Figure 8:**
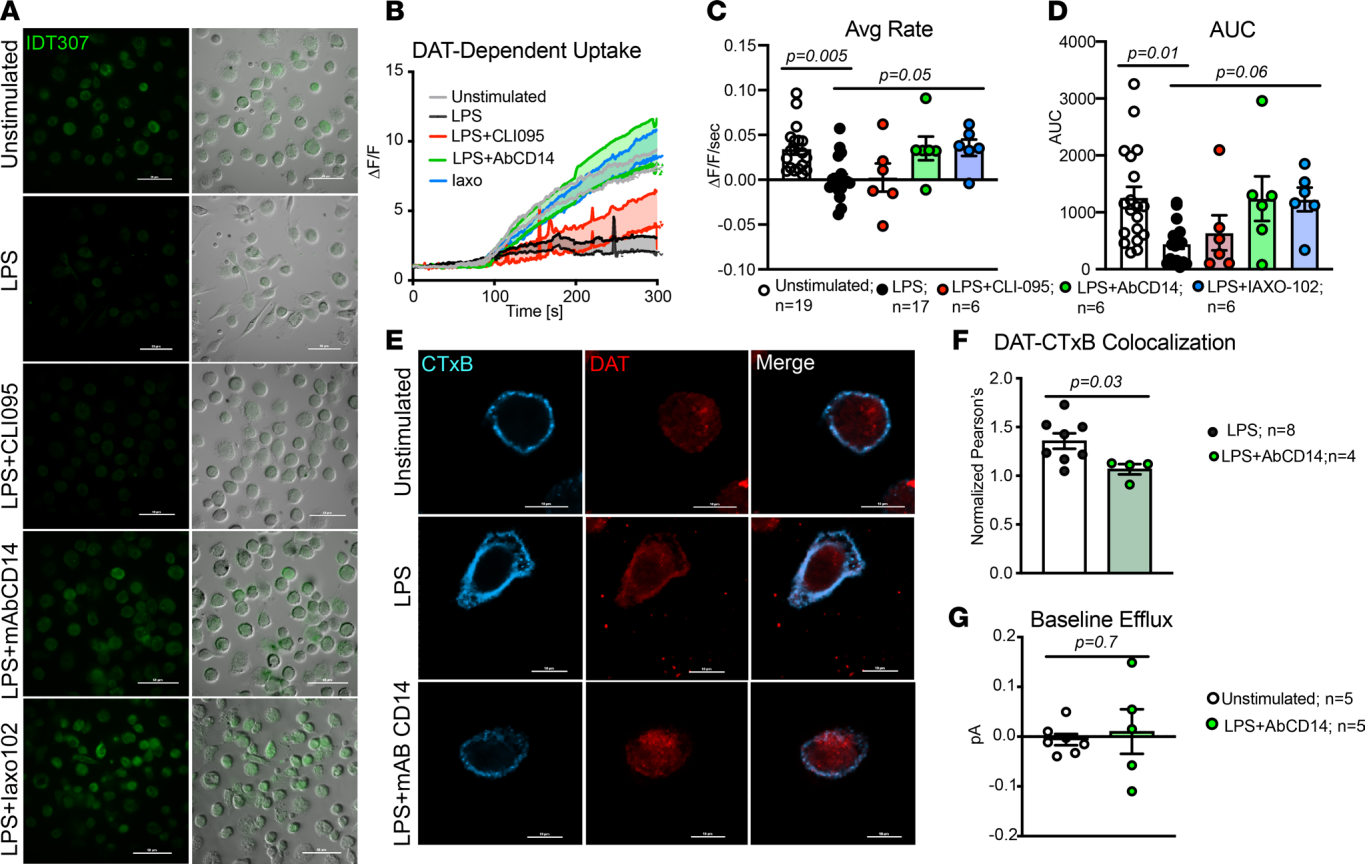
LPS-regulation of DAT activity is CD14 dependent but TLR4 independent. (**A**) Representative 40× images of macrophages that were unstimulated, LPS-stimulated, or cotreated with LPS and CLI095 (TLR4 antagonist), LPS and a neutralizing monoclonal antibody against CD14 (AbCD14), or LPS and Iaxo102 (a CD14 antagonist) and assayed for DAT-mediated IDT307. (**B**) DAT-dependent IDT307 uptake was calculated via blocker subtraction showing decreased DAT-dependent uptake in LPS-stimulated macrophages. Cotreatment with LPS and either AbCD14 or Iaxo102 prevented the LPS-induced reduction of DAT-dependent IDT307 uptake. (**C** and **D**) Quantification of rate (**C**, average slope) and magnitude (**D**, AUC) of DAT-dependent IDT307 uptake shown in **B** showed a significant decrease in the DAT-mediated IDT307 uptake following LPS stimulation (average slope, *P* = 0.005; AUC, *P* = 0.01). CLI095 did not prevent the LPS reduction of DAT-dependent uptake (AUC, *P* > 0.9), whereas cotreatment with either mAbCD14 or CD14 antagonist Iaxo102 rescued the DAT-dependent uptake back to unstimulated levels (average slope, *P* = 0.05; AUC, *P* = 0.06). Data are from *n* = 6–19 experiments/group, and statistical analysis was performed using Kruskal-Wallis tests with Dunn’s test for multiple comparisons. (**E**) Representative 60× images of cultured human macrophages that were either unstimulated, LPS-stimulated, or treated with LPS and mAbCD14 and labeled for CTxB-555 and DAT. (**F**) Quantifying colocalization between CTxB55 and DAT using Pearson’s correlation coefficient shows that AbCD14 reversed the increased DAT–CTxB-555 colocalization back to unstimulated levels (*P* = 0.03, Mann Whitney *U* test). Data in **E** and **F** are from *n* = 4–8 experiments/group. (**G**) The DAT-dependent dopamine efflux measured via simultaneous whole-cell patch-clamp and amperometry as in Figure 7 shows no significant increase in basal dopamine efflux between unstimulated macrophages and macrophages treated with LPS + mAbCD14 (*P* = 0.7, Welch’s 2-tailed *t* test, *n* = 5 experiments/group).

We next sought to elucidate the mechanism of (a) LPS-induced regulation of DAT activity and (b) how DAT-dependent dopamine efflux contributed to DAT’s potential immunomodulatory role. On macrophages, LPS initially binds CD14, which recruits TLR4, triggering a signaling cascade that activates NF-κB and induces an inflammatory response (54). Therefore, we hypothesized that either TLR4 or CD14 may mediate the LPS-induced regulation of DAT activity. This hypothesis was examined using 3 complementary approaches: an IDT307 uptake assay, DAT/GM1 localization, and measurement of DAT-dependent dopamine efflux in unstimulated and LPS-stimulated macrophages. The bath/vehicle solutions for all live-cell experiments contained NET and SERT antagonists. For the uptake assay, macrophages were treated with either vehicle (unstimulated), LPS, LPS + CLI095 (TLR4 antagonist, 3 μM), LPS + a neutralizing antibody against CD14 (AbCD14), or LPS + Iaxo102 (CD14 antagonist (Iaxo102, 5 μM). Cells were treated and assayed for DAT-dependent uptake as above (Figure 8, A and B). We should note that LPS treatment for 6 hours produced a similar amount of DAT-dependent IDT307 uptake as did a 24-hour treatment (Supplemental Figure 7, A and B; *P* = 0.4).

Consistent with the data shown in Figure 6, LPS inhibited DAT-dependent IDT307 uptake (Figure 8, C and D; unstimulated versus LPS, average slope, *P* = 0.005; AUC, *P* = 0.01). Blocking CD14 abrogated this effect, returning both the magnitude and the rate of uptake to levels seen in unstimulated macrophages (Figure 8, C and D) (LPS versus LPS + Iaxo102, average slope [*P* = 0.05], AUC [*P* = 0.06]; unstimulated versus LPS + AbCD14, average slope [*P* > 0.9], AUC [*P* > 0.9]; unstimulated versus LPS + Iaxo102, average slope [*P* > 0.9], AUC [*P* > 0.9]). The LPS-induced decrease in IDT307 uptake was unaffected by TLR4 inhibition (Figure 8, C and D; LPS versus LPS + CLI095, average slope, *P* > 0.9; AUC, *P* > 0.9). The lack of effect was not due to altered TLR4 and CLI095 binding, as concurrent treatment with this compound did block the LPS-induced increase in IL-6 production (Supplemental Figure 7B, *P* = 0.006).

Based on these results, we examined the effects of CD14 blockade on the LPS-mediated increase in DAT localization to GM1-enriched areas of the plasma membrane. Analysis of CTxB-555 and DAT colocalization in vehicle-, LPS-, and LPS + AbCD14–treated macrophages showed that treatment with AbCD14 significantly decreased DAT–CTxB-555 colocalization (Figure 8, E and F; *P* = 0.03). This suggests that blockade of CD14 decreased the LPS-induced localization of DAT to the GM1-enriched regions of the plasma membrane. Furthermore, basal DAT-dependent efflux was not different between unstimulated macrophages and macrophages treated with LPS + AbCD14 (Figure 8G; *P* = 0.7). Taken together, these data are consistent with the interpretation that LPS regulation of DAT on macrophages is CD14 dependent.

**Figure 9:**
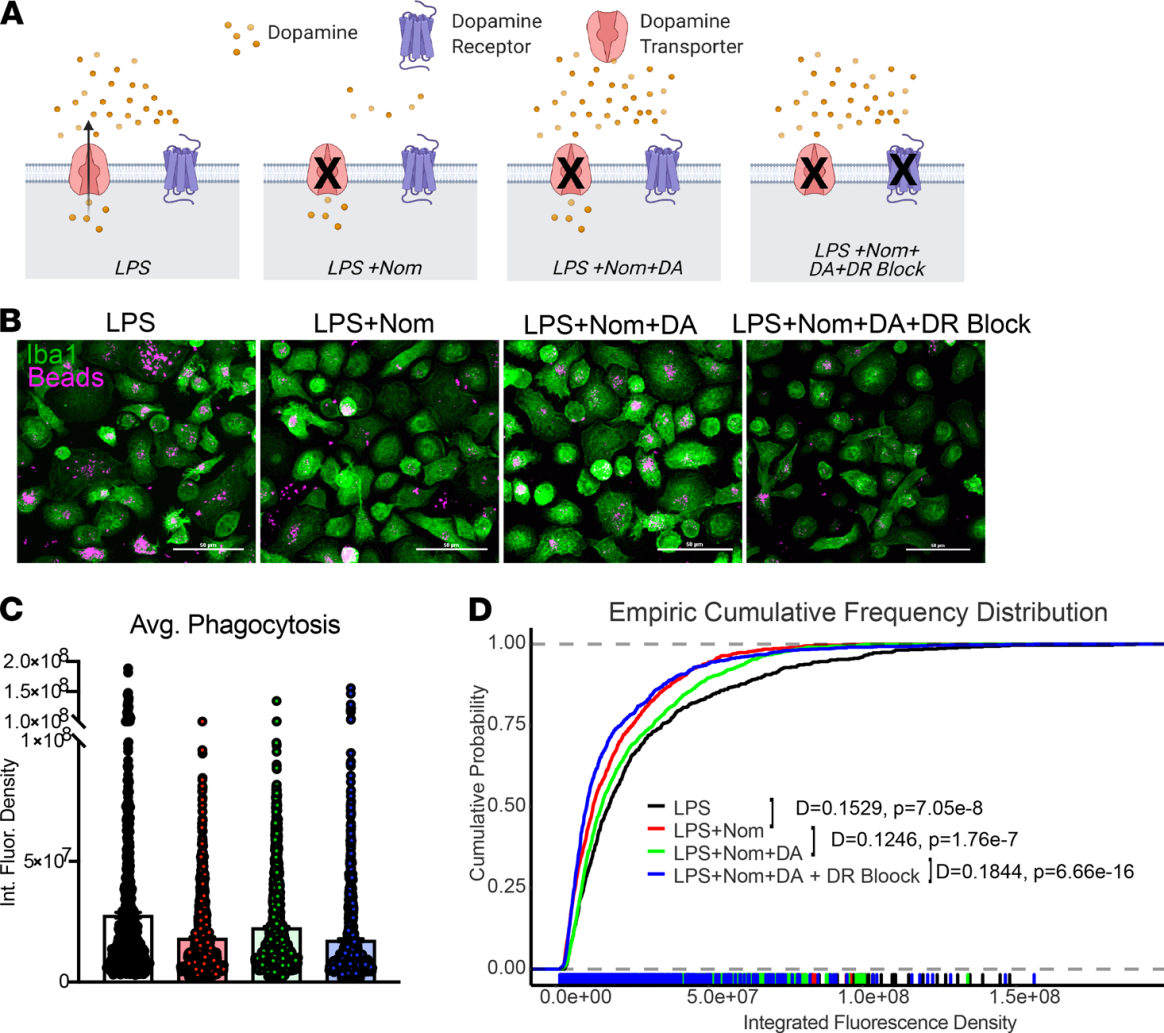
An autocrine/paracrine dopamine signaling loop could be an underlying mechanism for DAT modulation of human macrophages. (**A**) Schematic description of experimental design. Cultured human macrophages were treated with vehicle (unstimulated), LPS to induce dopamine efflux (increased extracellular dopamine), LPS + nomifensine to block DA efflux (decreased extracellular dopamine), LPS + nomifensine + exogenous dopamine, or LPS + nomifensine + exogenous dopamine + dopamine receptor blockade (Sulpiride and SCH53390). (**B**) Representative confocal images of PFA fixed cultured human macrophages incubated with fluorescent latex beads to measure phagocytosis under experimental conditions described in **A** (images for unstimulated condition not shown). (**C**) Median phagocytic capacity measured as fluorescence intensity of phagocytic beads/cell. (**D**) Empiric cumulative frequency distribution curves of phagocytic capacity shows that LPS + nomifensine decreases phagocytosis compared with LPS-stimulated macrophages (D-statistic = 0.1529, *P* = 7 × 10^–8^). Increasing extracellular dopamine by adding exogenous dopamine shifted the distribution curve to the right toward the LPS group representing an increase in phagocytosis (D-statistic = 0.1246, *P* = 2 × 10^–7^). Blocking both D1-like and D2-like receptors reversed the effect of extracellular dopamine, shifting the distribution curve back to the left toward the LPS + nomifensine curve, representing a decrease in phagocytosis compared with LPS + nomifensine + dopamine (D-statistic = 0.1844, *P* = 7 × 10^–16^). Images and data from **B**–**D** are from *n* = 596–1153 cells/group from at least 3 experiments/group.

Our findings indicate that (a) DAT dynamically regulates the availability of dopamine in a macrophage’s immediate microenvironment via uptake or, during LPS- induced inflammation, via dopamine efflux (Figure 3, Figure 6, and Figure 7); and (b) DAT is a potential immunomodulator of the macrophage response to LPS (Figure 5). These findings corroborate previous reports that macrophages contain the requisite machinery for dopamine synthesis and dopamine signaling, which has been shown to regulate macrophage immune functions (14). Nonneuronal cell types have recently been shown to release dopamine and signal onto their own or neighboring dopamine receptors (55). Additionally, immune cells have been shown to engage in autocrine/paracrine signaling for a variety of neurotransmitters (56). Hence, a switch from DAT-mediated removal of dopamine from the extracellular milieu to DAT-mediated release of dopamine to the extracellular milieu may engage dopamine receptors to mediate DAT’s immunomodulatory effects via an autocrine or paracrine loop. To test this, macrophages were exposed to 4 distinct treatments designed to pharmacologically dissect our proposed autocrine/paracrine loop (Figure 9A); (a) LPS treatment to increase DAT-mediated dopamine efflux and extracellular dopamine; (b) LPS treatment while blocking DAT to decrease extracellular dopamine (condition 1); (c) LPS treatment while blocking DAT and adding dopamine extracellularly, which we hypothesized would reverse the effect of DAT blockade; and (d) subsequently blocking dopamine receptors, which we hypothesized would resemble LPS with DAT blockade (condition 2). All conditions were tested in the presence of NET blockade. The macrophage response to each condition was then evaluated by measuring phagocytic activity, enabling evaluation of the effects of DAT-mediated dopamine release on a classical macrophage function (Figure 9B).

Quantifying the amount of phagocytosis by measuring the amount of fluorescence within each macrophage showed that blocking DAT decreased phagocytic capacity compared with LPS alone (Figure 9, C and D; D-statistic = 0.1529, *P* = 7 × 10^–8^), as shown previously (Figure 5, B–E). Macrophages from condition 3, which restored dopamine signaling by adding exogenous dopamine, showed a reversal of the effects of DAT blockade and increased phagocytic capacity approximating the LPS alone condition (LPS + Nom versus LPS + Nom + DA; D-statistic = 0.1246, *P* = 2 × 10^–7^). Macrophages from condition 4, which inhibited the exogenous dopamine signaling by blocking dopamine receptors, showed decreased macrophage phagocytosis approximating the levels seen in cells treated with LPS + Nom (D-statistic = 0.1844, *P* = 7 × 10^–16^) indicating dopamine’s effect on phagocytosis was specific to dopamine receptors (Table 1). Collectively, these findings suggest that DAT-mediated dopamine efflux increases extracellular dopamine signaling to modulate macrophage phagocytosis during LPS stimulation. They support the hypothesis that LPS stimulation of human macrophages induces a CD14-dependent shift of DAT activity favoring an efflux promoting conformation, which subsequently engages an autocrine/paracrine dopamine loop to modulate macrophage immune response to LPS.

## Discussion

MDMs are a critical population of innate immune cells with both protective and proinflammatory roles in a variety of tissues. Over the past 2 decades, an increasing number of studies suggested that biogenic monoamines, such as norepinephrine and dopamine, have immunomodulatory effects on human macrophages (8, 14). In addition to adrenergic and dopaminergic receptors, the NET and DAT are also expressed on macrophages (10, 24, 57). While most research to date has focused on receptor-mediated signaling by adding exogenous norepinephrine or dopamine, a recent study has shown that NET is a potent regulator of the phenotype of sympathetic neuron–associated adipose macrophages and is implicated in obesity (10), showing that biogenic amine transporters can regulate macrophage functions. Our findings support an immunomodulatory role for DAT, through multiple complementary approaches. Although data shown in this study are obtained from MDMs collected from total of 29 human subjects — thus, a limited number of samples in each experiment — the data across experiments are consistent. Overall, these data offer insights into how DAT activity may modulate macrophage-mediated immunity.

### The identity of DAT^+^ macrophages.

In the present study, we first observed the expression of NET and DAT on circulating human monocytes, cultured MDMs, and a subpopulation of intestinal macrophages in situ. During steady state, MDMs contribute heavily to the macrophage pools in gut and dermis and, to a lesser extent, in other tissues like lung and heart (1). During inflammation, or following resident macrophage depletion, MDMs will repopulate most niches, making MDMs physiologically relevant to study (2). A limitation of our cultured macrophages, however, is that they do not entirely recapitulate the molecular identity of macrophages in vivo (30, 58) and serve primarily to study fundamental macrophage biology. Nevertheless, the use of primary human macrophages provides a reliable human-based model system. Therefore, while we cannot say under which circumstances engrafted MDMs maintain DAT expression, we found consistent DAT expression in our human model system, and it is likely that at least some MDMs express DAT in either the steady state or under inflammatory conditions in tissue. This is supported by our observation that a subset of intestinal macrophages was DAT^+^, confirming some physiological relevance to DAT expression on macrophages. The scRNA-Seq data set from human colon further confirmed that the DAT transcript was expressed in a cluster consistent with tolerizing macrophages. Notably, the scRNA-Seq has limited resolution due to the diverse sample population (not sorted). The gut wall harbors a multitude of distinct macrophage subsets that vary based on their niche — that is, nerve-associated, blood vessel-associated (34), and lymphoid patch–associated (35), as well as tolerizing macrophages (36). Our data confirm that some macrophages, mostly in the submucosa, express DAT, but these data do not elucidate the precise phenotype or ontogeny of DAT^+^ macrophages. As DAT^+^ macrophages mostly localize around MAP2^+^ or lymphoid follicle regions, future studies will examine more precisely the complete signature and ontogeny of DAT^+^ macrophages in these regions. In addition, recent advances in CyTOF and RNA-Seq may provide more insights into DAT expression on macrophages of other tissues, identifying the specific transcriptomic identities that correlate with DAT expression.

### The role of macrophage DAT in the steady state.

Our study examined the baseline function of NET and DAT on cultured MDMs and found them to be capable of uptake. It is notable that there was heterogeneity in both DAT expression (Figure 1C) and activity (Figure 3, Figure 6, and Figure 8) across human donors. Future investigations may reveal the epigenetic and posttranslational mechanisms that can regulate DAT expression and activity on healthy human immune cells. Nevertheless, expression and activity were consistently detected, albeit with varying magnitudes. As biogenic monoamine transporters provide tight regulation of monoamine tone, our data support the notion that NET and DAT on macrophages help control the monoamine concentration in the proximal microenvironment of each macrophage. Because these experiments were done in vitro, we can say with certainty that DAT-dependent uptake is mediated by macrophages and not neurons or other cells.

Conversely, a limitation of the in vitro approach is that macrophages do not exist in isolation in vivo but interact extensively with neighboring cells, making it difficult to determine precisely how macrophage uptake of dopamine would affect the local environment. However, some tissue environments rich in macrophages, such as the gut, are also rich in dopamine. It is conceivable that the DAT-mediated uptake on macrophages may help regulate the overall dopaminergic tone of their niche. Indeed, here we show that some gut macrophages are DAT^+^, and such a role has already been shown for NET in adipose macrophages (10). The notion that macrophages can modulate extracellular dopamine content via the DAT-dependent uptake at rest is potentially novel and represents a foundation for further investigation. Such studies may use conditional and inducible knockouts specific to macrophages, e.g., CX3CR1-CreER mice, which will advance knowledge of how macrophage DAT contributes to baseline tissue functioning.

### Bidirectional regulation between DAT and immunity.

The central findings of this study are: (a) LPS stimulation induces DAT-dependent dopamine efflux and (b) DAT activity modulates the inflammatory response to LPS stimulation. This study used LPS as a potent inflammatory stimulus since increased LPS in the gut is associated with a variety of diseases. However, LPS is not the only stimulus that can evoke a macrophage response. IFNs, ILs, and a host of other pathogen-associated molecular patterns (PAMPs) and damage-associated molecular patterns (DAMPs) bind to macrophage receptors (59). Further complicating this picture are the intricate mechanisms regulating DAT activity by affecting trafficking (40, 60), multimer formation (61, 62), conformational state (63, 64), membrane potential (20, 65–68), and transport kinetics (69, 70). Indeed, here we show that one classic mechanism of DAT regulation, PMA-induced internalization, is preserved in macrophages, and it is likely that others are, as well. Moreover, given their different signaling pathways, it is highly probable that different immune signals have divergent effects on DAT activity.

Why study DAT activity on macrophages in different paradigms? Our data imply that DAT may play a role in downmodulating the inflammatory response. Inhibiting DAT during LPS stimulation skewed the macrophage phenotype to a more proinflammatory state characterized by increased cytokine production and decreased phagocytosis. This was due to the efflux of dopamine that can increase local dopaminergic tone and enhance dopamine receptor signaling. An increase in DAT-mediated dopamine release is also consistent with a previous report showing that LPS upregulates catecholamine synthesis (71). However, it is important to consider the nuances of DAT regulation of dopamine homeostasis. It follows that DAT’s immunomodulatory properties may also be influenced by mechanisms mediated by other factors — e.g., intracellular dopamine. Cytosolic dopamine can induce oxidative stress (72), which is consistent with the elevated mitochondrial superoxide species observed in our study. Mitochondrial function and metabolism are potent regulators of myeloid phenotype (38, 39, 73). Alternatively, it has been proposed that cytosolic dopamine may enter the nucleus and regulate transcription (74, 75). Additionally, macrophages may exhibit vesicular dopamine release, as lymphocytes exhibit a partially calcium-dependent release of norepinephrine (76), adding another variable in local dopamine homeostasis. While DAT plays a major role in the regulation of local dopaminergic tone, the network of mechanisms controlling dopamine inside and outside of the macrophage are likely a composite of the above, and further studies utilizing technologies like fluorescent dopamine sensors (77) are needed to provide clarity.

Irrespective of the intricacies above, our findings indicate DAT’s potential to modulate immunity. These data also corroborate a previous study showing increased proinflammatory cytokine production from splenic macrophages in DAT^–/–^ mice (78). While these data provide a limited glimpse into macrophage functions, they raise the question of whether other macrophage functions, such as antigen presentation or chemotaxis, are affected by endogenous dopamine regulation. Indeed, dopamine receptor activation on T cells during antigen presentation was associated with increased activation (79). It is tempting to ask how DAT function may also affect more specialized macrophage functions, such as neuronal support in the gut (9, 34). The current study provides the potential groundwork to continue studying the relevance of DAT in immunity. Future studies could employ coculture systems and in vivo tools such as fluorescent reporters for T cell activation (80) to unravel further roles for the various modes of DAT activity on macrophages.

### Summary and conclusion.

This study provides an in-depth functional characterization of both NET and DAT on human macrophages. We introduce a potentially novel, bidirectional regulation between macrophage DAT and immunity, mediated by LPS-induced dopamine efflux that enhances an autocrine dopamine loop. Critically, this indicates that DAT may differentially regulate dopamine concentrations in the proximal microenvironment in response to distinct stimuli, triggering changes in macrophage function and suggesting an active role for DAT in the macrophage immune response. Overall, the findings of this study support an important regulatory role for DAT in the innate immune response and should serve as a call for further examination of macrophage DAT in inflammatory and autoimmune settings.

## Methods

Supplemental Methods are available online with this article.

### Statistics.

Statistical analysis was performed in GraphPad Prism or in R. For specific statistical tests and post hoc tests, see figure legends. Further details can be found in Supplemental Methods.

### Study approval.

Regarding human blood samples, this study was approved by the University of Florida’s IRB (no. 201701195). To obtain macrophages of healthy donors, blood samples were purchased from LifeSouth Community Blood Center (Gainesville, Florida, USA). As outlined in the Supplemental questionnaire 1, the donors were healthy individuals aged 20–70 years old, either male or female; they were not known to have any bloodborne pathogens, and they were never diagnosed with a blood disease such as leukemia or bleeding disorders. None of the donors were using any medications for an infection, nor were they on any blood thinners. Whole blood samples from patients with Parkinson’s disease were obtained via an IRB-approved protocol (no. 201701195). Following the informed consent process, patients diagnosed with Parkinson’s disease by a board-certified movement disorders physician and age-matched healthy controls had 20–30 mL of blood drawn. Whole blood samples were then immediately taken for processing and monocyte isolation.

Formaldehyde-fixed, paraffin-embedded samples of human colon were obtained via an IRB-approved protocol (no. 202002059) from the UF Center for Translational Science Institute Biorepository under a confidentiality agreement.

## Author contributions

Data collection and analysis were contributed by PMM, AG, DMM, AN, RAN, KR, SMM, JM, JER, and KA. Study conception was contributed by PMM, ARZ, PJG, MSO, WJS, and HK. Experimental design and optimization were contributed by PMM, AG, AD, LY, PJG, and HK. Manuscript preparation was contributed by PMM and HK. Manuscript revision was contributed by PMM, AG, ARZ, MSO, WJS, and HK.

## Supplementary Material

Supplemental data

Supplemental video 1

## Figures and Tables

**Table 1:**
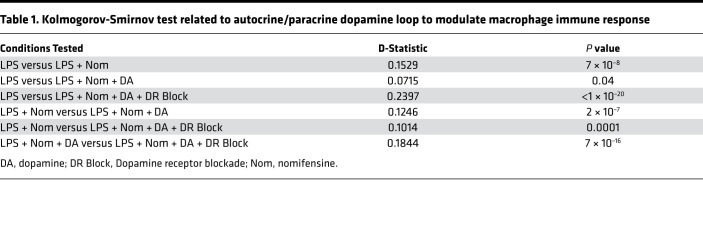
Kolmogorov-Smirnov test related to autocrine/paracrine dopamine loop to modulate macrophage immune response

## Version 1 01/11/2022

In-Press Preview

## Version 2 02/22/2022

Electronic publication
