# Supplementary material for: Functional characterization of the biogenic amine transporters on human macrophages
Source: JCI Insight. 2022 Feb 22;7(4):e151892. doi: 10.1172/jci.insight.151892 (PMC8876465; doi:10.1172/jci.insight.151892)
Supplement: Supplemental data [file jciinsight-7-151892-s209.pdf]

Supplemental Figure 1

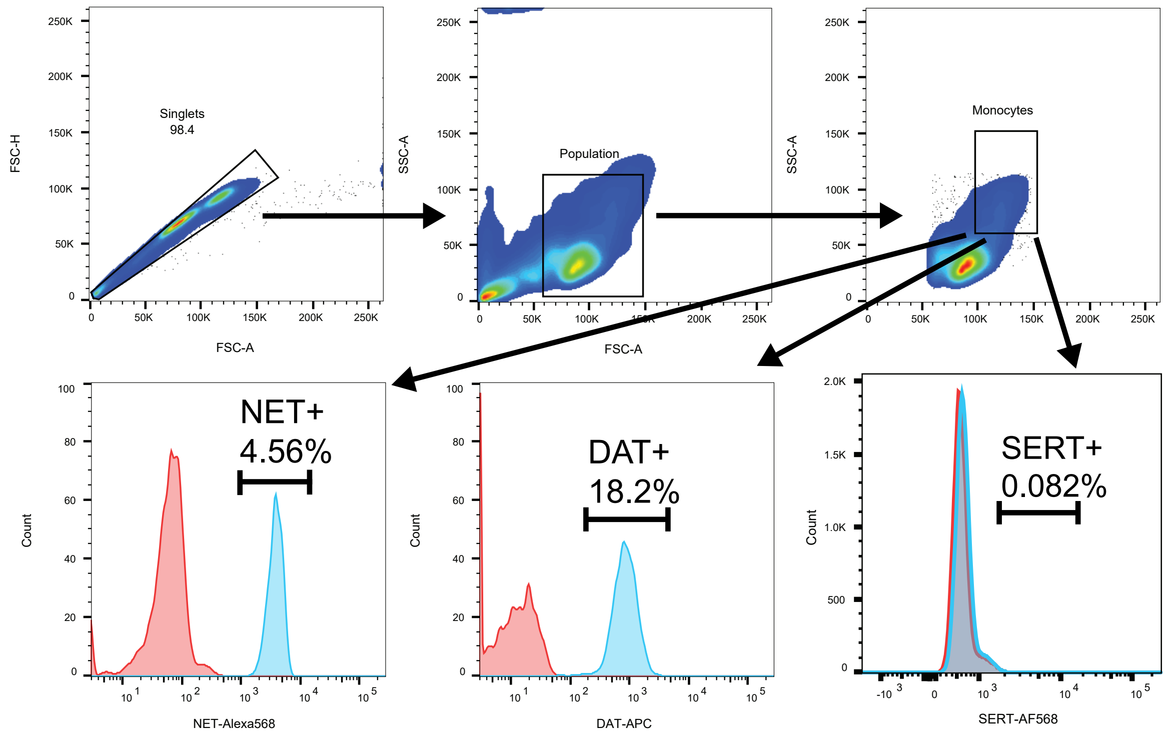

Supplemental Figure 2

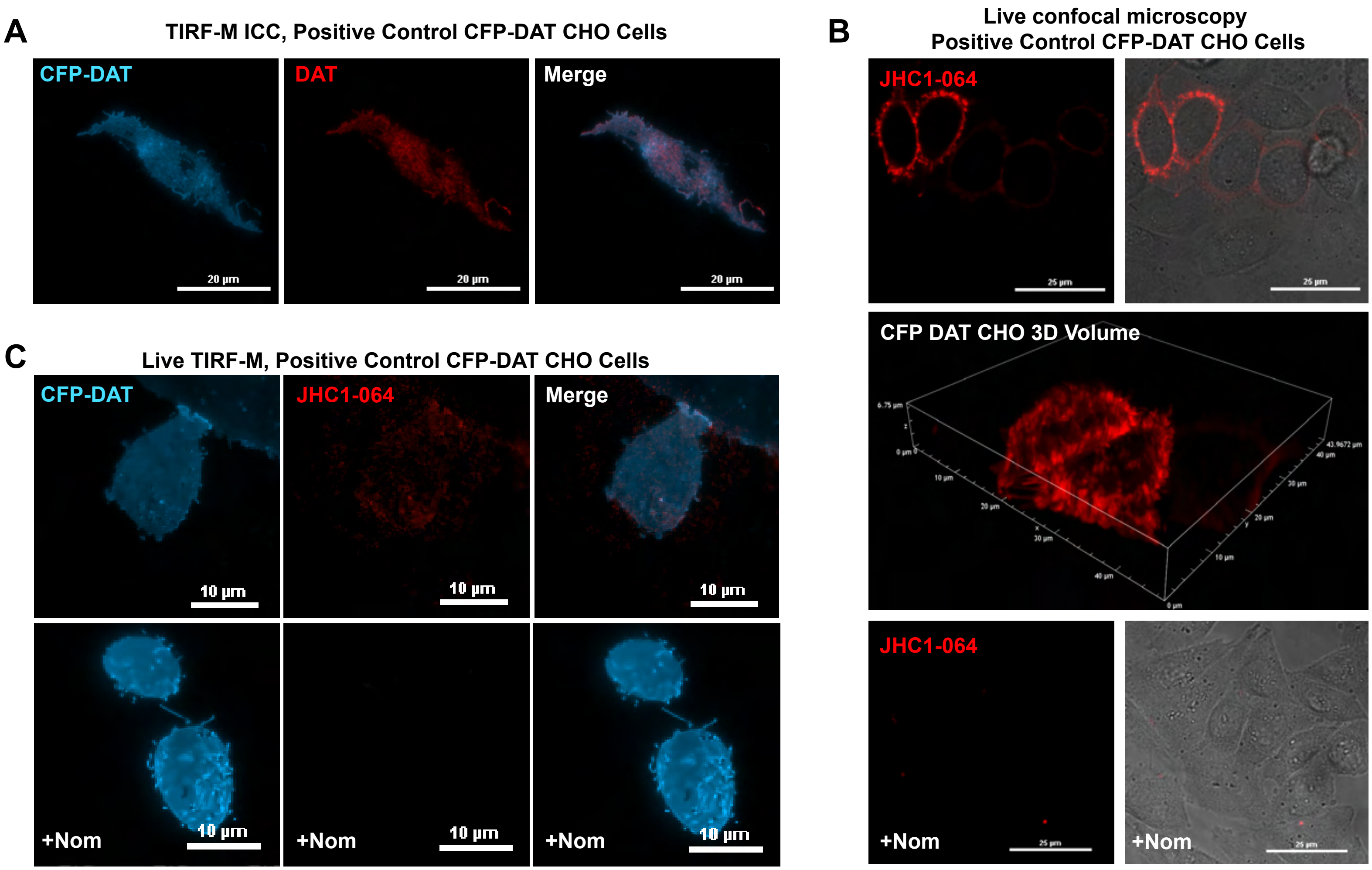

Supplemental Figure 3

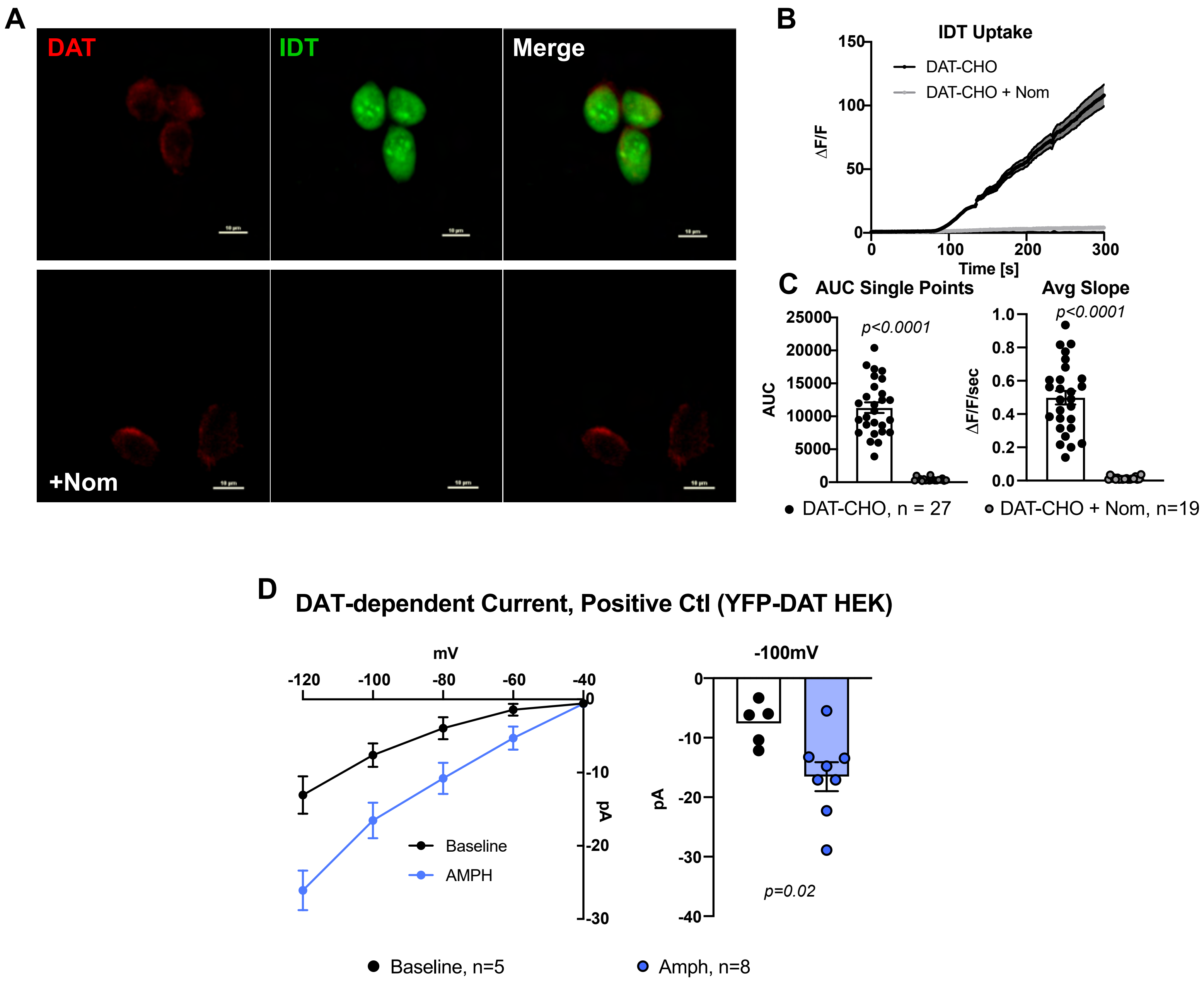

Supplemenal Figure 4

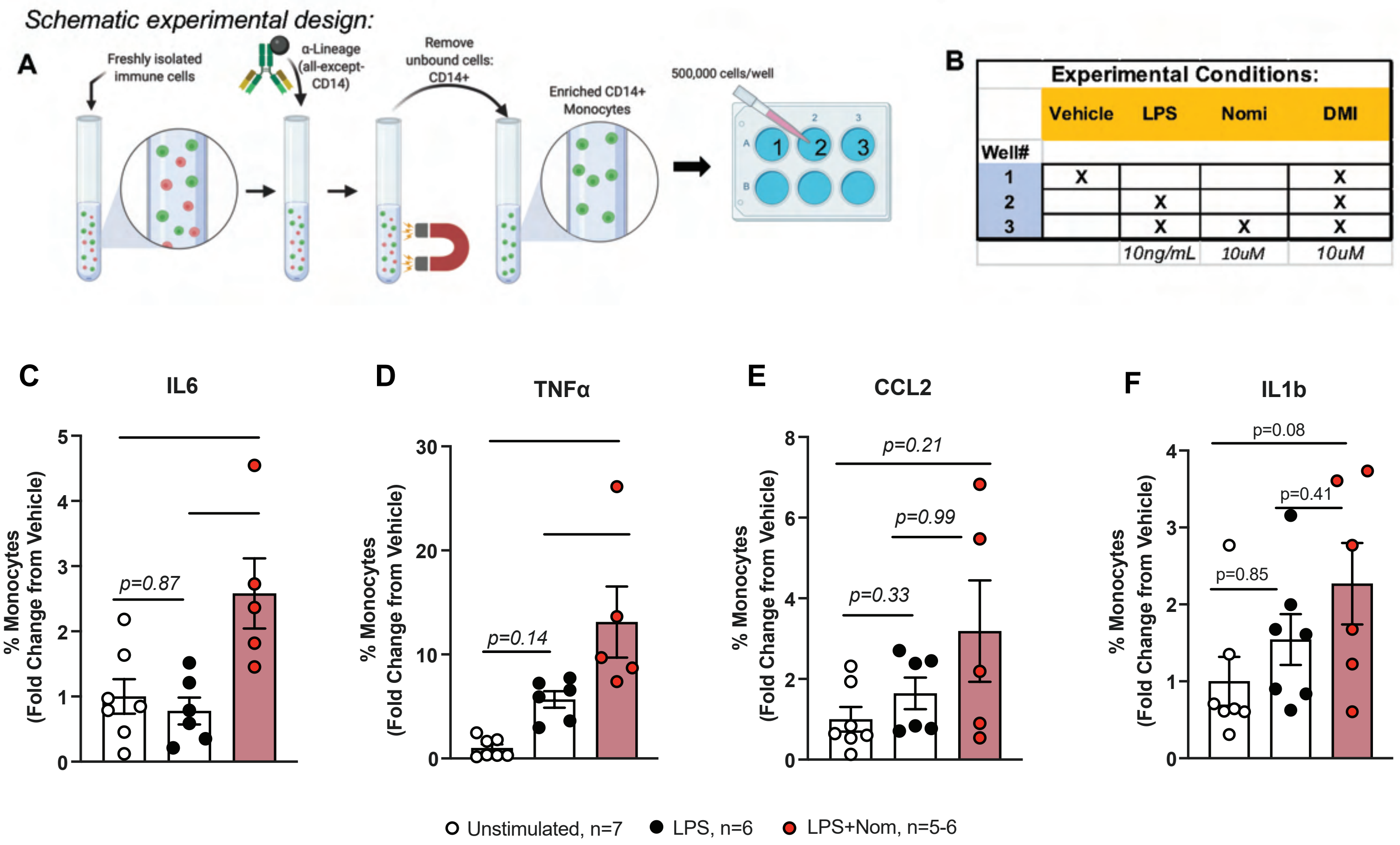

Supplemental Figure 5

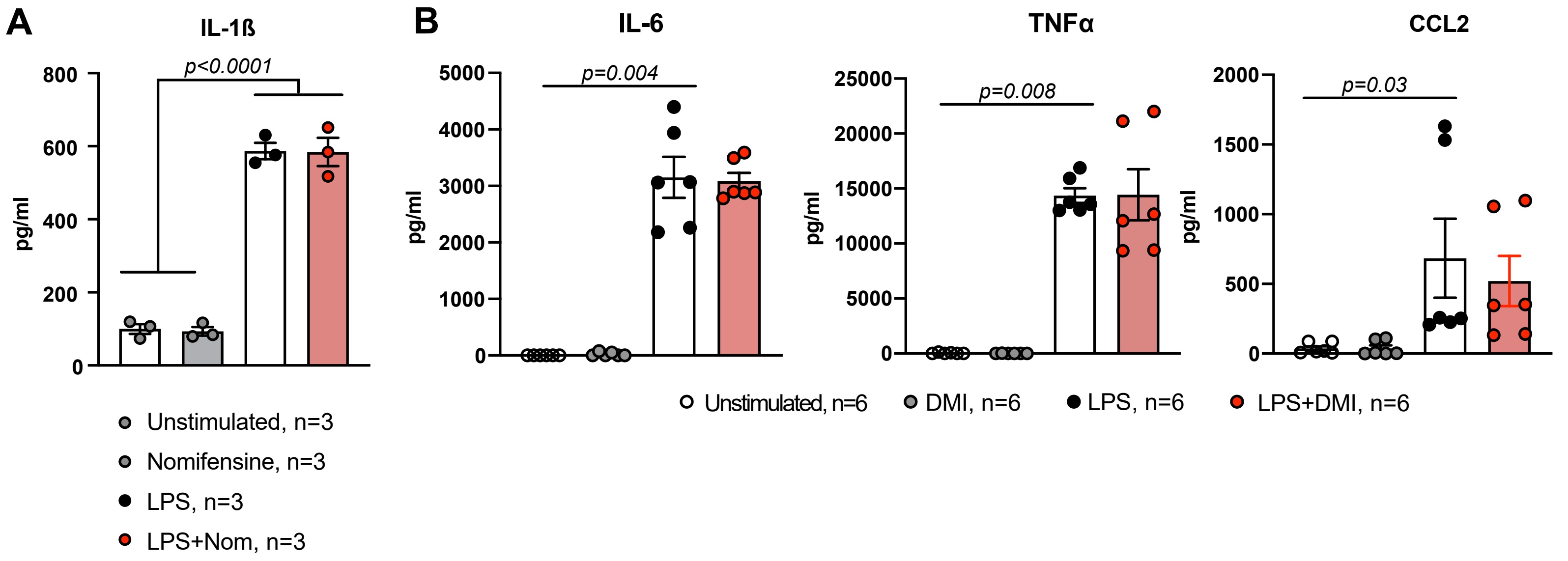

Supplemetal Figure 6

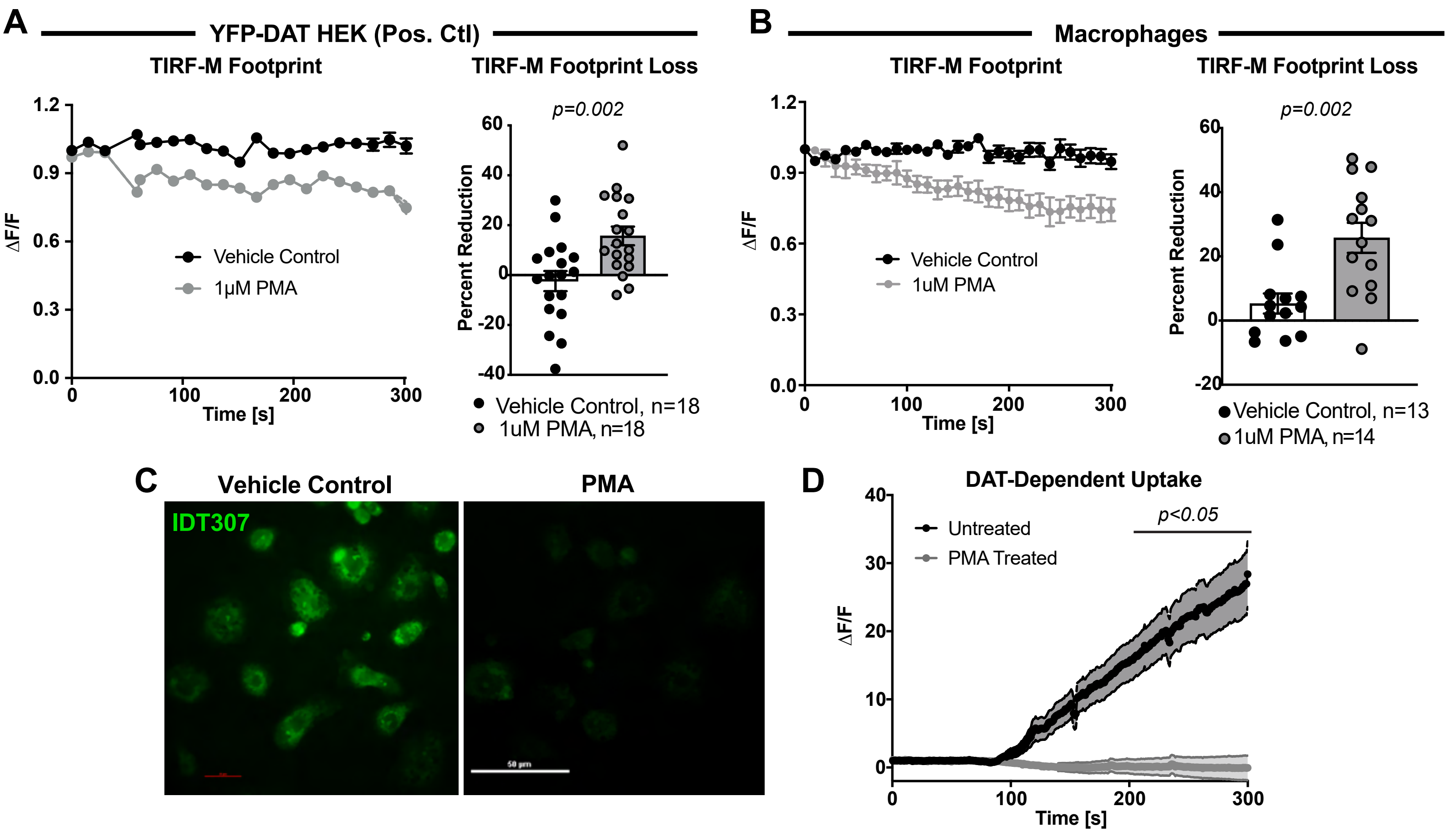

Supplemenal Figure 7

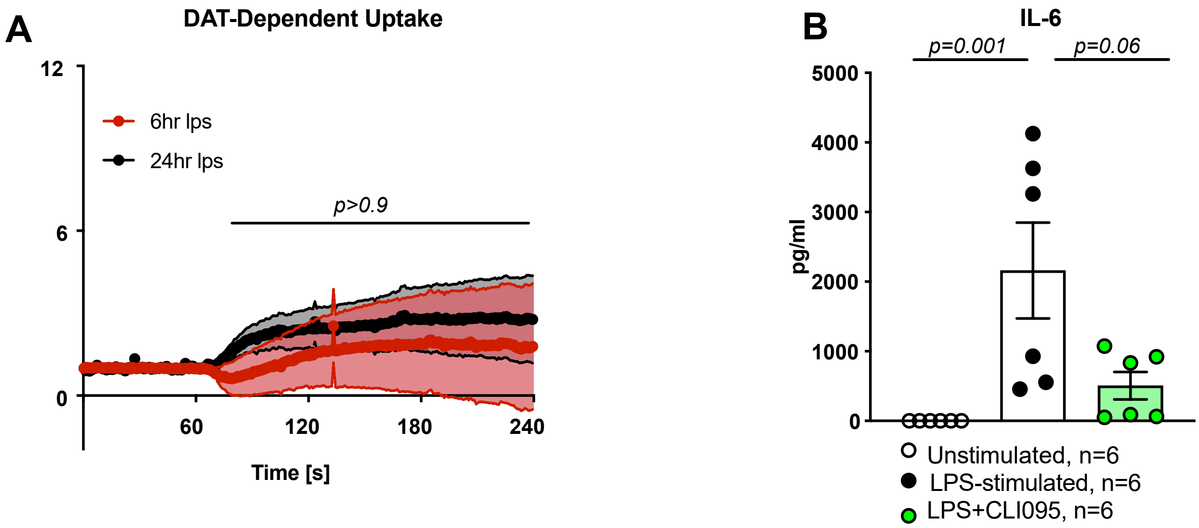

Supplemental Figure 1: Gating scheme for flow cytometry in Figure 1. Whole peripheral blood mononuclear cells were isolated from human blood and assayed for NET and DAT expression via flow cytometry. Doublets were excluded on the basis of FSC-H and FSC-W. Monocytes were identified from the singlet population on the basis of FSC-A and SSC-A. NET- and DAT-expressing monocytes were identified from the monocyte population on the basis of NET-Alexa568 fluorescence or DAT-APC fluorescence, respectively.

Supplemental Figure 2: Validation of assays for detection of NET and DAT. (A) Representative images of CFP-tagged-DAT-expressing CHO cells (CFP-DAT CHO) were fixed, labelled with an anti-DAT antibody, and imaged using TIRF microscopy, showing overlap between the CFP tag and the DAT antibody labelling. (B) Representative images of CFP-DAT CHO cells incubated with JHC1-064 and monitored for binding under live-cell confocal microscopy in the absence (top) or presence (bottom) of DAT blockade (Nomifensine). No JHC1-064 fluorescence was observed when DAT was blocked. (C) Representative TIRF-M images of CFP-DAT CHO cells following JHC1-064 binding in the absence (top) or presence (bottom) of DAT blockade (Nomifensine). No JHC1-064 fluorescence was observed on CFP+ cells in the presence of DAT blockade (+nomifensine). Images are from at least 3 experiments/group.

Supplemental Figure 3: Validation of assays for functional characterization of NET and DAT. (A) Representative images of CFP-DAT CHO cells assayed for DAT-mediated uptake using IDT307 perfusion in the absence (top) or presence (bottom) of DAT blockade (Nomifensine). (B) The DAT-mediated IDT307 uptake measured as change in intracellular fluorescence above baseline ($\Delta F/F$), revealing appreciable IDT307 uptake in the absence of DAT-blockade. (C) Nomifensine significantly inhibited the magnitude (AUC, left, $p<0.0001$, unpaired t-test) and rate (Average Slope, right, $p<0.0001$, unpaired t-test) of DAT-mediated IDT703 uptake, indicating DAT-specificity. Images and data are from 19-27 CFP+ cells over 3 experiments. (D) HEK cells stably expressed YFP-tagged DAT (YFP-DAT HEK cells) were patch clamped in whole cell configuration under voltage clamp mode, and inward currents were evoked by serial hyperpolarization steps. DAT-dependent current was calculated by subtracting the inward currents in the presence of DAT blockade from the inward currents at baseline or in the presence of DAT-activator amphetamine (Amph). Amphetamine significantly increased the DAT-dependent inward current ($p=0.0202$, unpaired t-test, n=5-8)

Supplemental Figure 4: DAT inhibition enhances pro-inflammatory intracellular cytokine expression and release in acutely isolated monocytes. A) Schematic diagram describes the experimental design: Enriched CD14+ monocytes were seeded into 6-well ultra-low adherence plates at 500,000 cells/well, containing RPMI plus 7.5% heat inactivated autologous serum. B) Table outlines experimental groups: Cells were treated with vehicle, LPS (10ng/mL) or nomifensine (10uM). To isolate dopamine receptor or DAT specific effects, all experiments were performed in the presence of NET antagonist desipramine (DMI, 10uM). C-F) LPS treatment in the presence of Nomifensine significantly increases intracellular IL6 and TNFα (C & E, $p<0.05$, $p<0.001$, respectively),

and both intracellular CCL2 and IL1b show similar trends (D & F). Statistical analysis was performed using one-way ANOVA with Tukey's correction for multiple comparisons.

Supplemental Figure 5: DAT inhibition has no effect on LPS-induced IL-1β, and NET inhibition does not affect LPS-induced cytokine release. (A) Lysates from macrophages treated with vehicle, nomifensine, LPS, or LPS+Nomifensine were measured for IL-1β expression via ELISA. LPS significantly increased the levels of IL-1β ($p<0.0001$) and LPS+Nomifensine had no further effect ($p>0.9$); n=3 experiments/group. Data analyzed via one-way ANOVA with Tukey's test for multiple comparisons (B) Conditioned media from macrophages treated with vehicle (unstimulated, n=6), Desipramine (DMI, n=6), LPS (n=6), or LPS+DMI (n=6) was assayed for IL-6 (left), TNFα (middle), and CCL2 (right). LPS induced significant increase in release of pro-inflammatory cytokines and blockade of NET with DMI had no additional effect. Statistical analysis was performed using a Kruskal-Wallis test with Dunn's test for multiple comparisons.

Supplemental Figure 6: DAT on macrophages is subject to canonical regulatory mechanisms. (A) HEK cells stably transfected with YFP-DAT were incubated with JHC1-064 then treated with 1µM PMA while the JHC1-064 signal at the membrane was recorded using TIRF microscopy. Quantifying the change in JHC1-064 fluorescence over time (left) indicated a significant reduction in the JHC1-064 TIRF-M Footprint in the PMA-treated cells compared to vehicle control ($p=0.002$, Mann-Whitney test, n=18/group). (B) Macrophages were incubated with JHC1-064 and treated with 1µM PMA while the JHC1-064 signal was recorded using TIRF microscopy. Quantifying the change in fluorescence over time (left) revealed a significant reduction in the JHC1-064 TIRF-M footprint in the PMA-treated macrophages compared to vehicle control ($p=0.002$, Mann-Whitney test, n=13-14/group). (C) Representative images of macrophages pre-treated with either vehicle or 1µM PMA then perfused with IDT to assess uptake. (D) Blocker subtraction was performed as previously described to isolate the DAT-dependent IDT307 uptake ($p<0.05$ from 214s-300s, two-way ANOVA with Sidak's test for multiple comparisons).

Supplemental Figure 7: 6 and 24 hour LPS-stimulated macrophages exhibit similar DAT uptake, and validation of CLI095. (A) Macrophages were treated for either 6 or 24 hours with LPS and assayed for DAT-dependent IDT30 uptake calculated via blocker subtraction. Uptake was not different between the two groups ($p>0.9$ for all time points, two-way ANOVA with Sidak's multiple comparison test). (B) Macrophages were treated with vehicle (unstimulated), LPS, or LPS + TLR4 inhibitor CLI095 and their conditioned media was assayed for IL-6 concentrations. LPS significantly increased IL6 release ($p=0.0058$) that was reduced with addition of CLI095 ($p=0.0323$, Kruskal-Wallis test with Dunn's test for multiple comparisons, n=6).

Supplemental Methods

Solutions and Reagents: Live-cell imaging experiments and electrophysiology experiments were performed using external solution containing 130mM NaCl, 10mM HEPES, 34mM Dextrose, 1.3mM KH_2PO_4, 1.5mM $CaCl_2 \cdot 2H_2O$, 0.5mM $MgSO_4 \cdot 7H_2O$,filtered (0.22μm sterile filter), adjusted to pH 7.4 and osmolarity between 270-300mOsm. Pipettes for electrophysiology/amperometry were filled with internal solution containing, 90mM CsCl, 0mM/30mM NaCl, 0.1mM $CaCl_2 \cdot H_2O$, 2mM $MgCl_2 \cdot 6H_2O$, 1.1mM EGTA,10mM HEPES, and 30mM Dextrose. To account for potential off-target effects due to norepinephrine and serotonin transporters, all experiments were performed in the presence of 1μM desipramine (DMI, Sigma Aldrich, St. Louis, MO), and 1μM fluoxetine (Flo, Tocris, Bristol, UK). To measure DAT-dependent effects, experiments included a DAT blockade condition using 5-10μM nomifensine (Nom, Sigma Aldrich, St. Louis, MO). The reagents' sources, solutions' compositions, analysis software, and the equipment employed for biochemical analysis, live or fixed cell confocal microscopy imaging, TIRF microscopy, electrophysiology and amperometry are outlined in Supplemental Tables 1, 2, and 3.

Human colon tissue: The colon samples were confirmed to be histologically normal by a board-certified pathologist. Blocks of tissue were cut into 5μm sections and mounted on slides, de-paraffinized, and rehydrated. Mounted tissue samples were immersed in Na-citrate buffer (10mM Na-Citrate, 0.05% Tween-20, pH to 6.0 with 1N HCl) then subjected to 24 hours of photobleaching under a broad-spectrum LED light at 4°C to reduce autofluorescence. Following photobleaching, antigen-retrieval was performed with the samples immersed in Na-citrate buffer and steamed at 95°C for 45 minutes. Tissue samples were blocked/permeabilized using 10% normal goat serum in 0.3% Triton-X in PBS for 3 hours at room temperature followed by additional avidin biotin blocking using a kit according to manufacturer's instructions (BioLegend, 927301). Primary immuno-staining was performed using antibodies: chicken anti-MAP2 (1:800, BioLegend, Poly28225), rabbit anti-IBA1 (1:800, Encor, RPCA-IBA1), and rat anti-DAT (1:200, Millipore, MAB369), in 5% normal goat serum 0.3% Triton-X in PBS overnight at 4°C. Slides were washed in PBS for 3x for 20 minutes each at room temperature with gentle shaking. Secondary immuno-staining was performed using: anti-chicken AlexaFluor-488+ (1:800, Invitrogen, A32931), anti-rabbit AlexaFluor-568 (1:800, Invitrogen, A-11011), and anti-rat biotin (1:200, Vector, BA-9401) for 1 hour at room temperature. Samples underwent another round of washes as above. Tertiary immuno-staining was performed using Streptavidin- 647 (1:200, Invitrogen, S-32357) for 1 hour at room temperature followed by another round was PBS washes, with the last wash going overnight. Slides were then counterstained with DAPI (1:5000, Invitrogen, D-1306) and mounted with Fluoromount Gold and allowed to dry for 1 day.

Immuno-stained colon samples were imaged using a Nikon A1 laser scanning confocal microscope (Nikon Instruments, Melville NY) and a x40 1.3 NA oil-immersion Plan-Apo objective. DAPI, MAP2, IBA1, and DAT signals were acquired via excitation with 405, 488, 561, and 640nm solid argon lasers and detected at 450/50, 525/50, 595/50, and 700/75nm, respectively. Channels were acquired in sequential series to minimize bleed-through. Z-stacks were acquired in 0.25μm steps through the sample at various anatomical locations in the colon wall and converted to maximum intensity projections. Further processing was performed in Nikon Elements NIS Analysis software (Nikon Instruments, Melville, NY).

Macrophage Isolation and Culturing: Macrophages were isolated from human blood using Ficoll density gradient separation as previously described [1]. Briefly, blood was diluted with 1x PBS 1:1 ratio and then overlaid on the Ficoll for a final volume ratio of 5:5:3 for blood: PBS: Ficoll, respectively. After centrifugation, the buffy coat layer was extracted, washed with PBS, re-suspended in Monocyte Adhesion Medium (MoAM) and then plated on either 12mm #1.5 round glass cover slips or glass- bottomed dishes, and incubated for 1-2 hours at 37°C with 5% CO_2 to allow monocytes to adhere. MoAM was made fresh for each culture using 7.5% autologous serum

in 1640 RPMI (Corning, Corning NY) without any supplements. After the incubation, plates and dishes were washed 3x with RPMI, given fresh MoAM, and returned to the incubator. Media changes were done 1 day and 3-4 days post-culture. All experiments were run on days 6 or 7, except where explicitly stated. The experiments were performed between 4-6 days after plating.

Flow cytometry for DAT NET and SERT: As previously published, we noted that freshly isolated PBMCs resulted in fewer than 0.5% dead cells during acquisition [2]; therefore, viability dye was not included in fresh PBMC samples. Every sample was stained in parallel with unstained, secondary only and isotype controls. Following PBMC isolation, cells were counted with trypan blue exclusion for viability, and density adjusted to 10,000 cells per microliter. 1 million cells per condition were aliquoted and immediately fixed for 30 minutes at room temperature (eBioscience Fix/perm kit, 88-8824-00), followed by washes in permeabilization buffer per manufacturer instructions. Primary antibodies against DAT (Sigma, MAB369, 1:100), NET (MabTechnologies, NET17-1, 1:100) and SERT (MabTechnologies, ST51-2, 1:100) were added and incubated for 30 minutes, followed by species-specific fluorochrome-conjugated secondaries (1:100 dilution). Data were acquired immediately following staining (Sony Spectral Analyzer, SP6800), and analyzed using FlowJo (Treestar).

Flow cytometry for intracellular cytokines: Monocytes were isolated from total PBMCs prepared as described above (Gopinath et al. 2020) using negative selection (Biolegend, 480048) per manufacturer's instructions. Total PBMCs were Fc-blocked to reduce nonspecific binding, followed by incubations with biotin-conjugated antibody cocktail containing antibodies against all subsets except CD14 (negative selection), followed by incubation with magnetic-Avidin beads, allowing all subsets other than CD14+ monocytes to be bound to the magnet. CD14+ monocytes were collected from the supernatant fraction, washed, counted and density adjusted such that 500,000 CD14+ monocytes were seeded per well and treated for 6 hours with vehicle, LPS (10ng/mL), or LPS plus Nomifensine (10uM) in an ultra-low-adherence 6-well plate (Corning, 3471) to prevent adherence. Suspended cells from each treatment group were aspirated and placed in a 15mL conical tube, with any remaining adherent cells detached by incubation with 700uL Accumax solution for 3 minutes (Innovative Cell Technologies, AM105) and added to suspended cells. After pelleting cells by centrifugation (3 minutes x 100g, room temperature), cells were assayed by flow cytometry.

As previously published [2], cells for flow cytometry were fixed and permeabilized (eBioscience, 88-8824-00), and stained for intracellular markers MCP1/CCL2 (Biolegend, 505909, APC, 1:100), TNF-alpha (Biolegend, 502908, PE,1:100), IL1beta (Biolegend, 511709, Pacific blue, 1:100), or IL6 (Biolegend, 501121, PE- Dazzle594, 1:100). Data were acquired immediately following staining (Sony Spectral Analyzer, SP6800), with compensation applied at the time of acquisition. Data were analyzed using FlowJo (Treestar).

RT-qPCR: Total RNA was extracted from cells using Trizol or the RNeasy Mini Plus™ kit (Qiagen), and RNA quantity and purity were determined by NanoDropOne spectrophotometer (Nanodrop Technologies). RNA (1 µg) was used to synthesize cDNA from each donor using the high-capacity reverse transcriptase cDNA synthesis kit (Abcam). DAT, and the housekeeping genes UBC, OAZ1, and TBP were amplified from cDNA by quantitative PCR (qPCR) on a QuantStudio 7 using gene-specific primers. TaqMan Fast Universal Master Mix, and PCR assay probes for DAT (Hs00997374_m1), 18s (4319413E), UBC (Hs00824723_m1), OAZ1 (Hs00427923_m1), and TBP(Hs00427620_m1) genes were purchased from Applied Biosystems (ThermoFisher, Waltham, MA, USA). A ratio of the 3 housekeeping genes was calculated by taking an average which was then subtracted from the DAT value to get a normalized CT DAT value. The normalized DAT value was then transformed using the equation 2-ΔCT. The

transformed value was then multiplied by 10,000 and plotted. Positive donors were those that expressed DAT in at least 1 of 3 wells tested.

LPS Stimulation and Blockades: Ultra-pure lipopolysaccharide from E. coli was added to a final concentration of 10ng/ml. Cells were cultured as described above, supplemented with 7.5% autologous serum, then either treated with fresh media (un- stimulated) or LPS-supplemented media (LPS-stimulated) and incubated at 37°C with 5% CO_2 for either 6 or 24 hours before beginning experiments. For mechanistic studies (Figure 8), the macrophages were pre-treated with 3μM CLI-095, a TLR4 antagonist [3] (Invitrogen, San Diego CA) for at least 1 hour (6hr LPS stimulation) or 3 hours (24hr stimulation). After pre-treatment, macrophages were treated with LPS+CLI-095 (Invitrogen, San Diego CA) at their respective concentrations for either 6 or 24 hours. Neutralizing CD14 antibody (10μg/ml, BioLegend) or a small molecule CD14 inhibitor, Iaxo-102 (5μM, AdiopoGen) was used in separate sets of experiments to test CD14- dependency of the measured responses. Macrophages were pre-treated with CD14 antibody for at least 1 hour(Kurt-Jones et al. 2000) before being treated with LPS+CD14 antibody following the same LPS-stimulation paradigm described above. For inhibitor alone control, the un-stimulated macrophages received antibody or CLI-095.

Biotinylation and Immunoblotting: Unstimulated and LPS-stimulated macrophages were washed three times with cold PBS, then incubated with sulfo-NHS-biotin (1.5mg/mL; ThermoFisher Pierce, 21331) for 30 minutes at 4°C while rocking. Remaining sulfo-NHS- biotin was quenched with cold Quenching Solution (glycine 50mM in PBS), followed by three washes with cold PBS. Cells were lysed in BufferD lysis buffer (10% glycerol, 125mM NaCl, 1mM EDTA, 1mM EGTA, pH 7.6) containing 1% TritonX and protease inhibitor cocktail (Millipore, 539131) for 1 hour at 4°C while rocking, followed by centrifugation for 15 minutes at 12,000g. The supernatants were divided into three portions: 25uL for protein quantification, 300uL for incubation with Avidin, with the remainder for whole lysate input. After equilibrating monomeric UltraLink Avidin (ThermoFisher Pierce, 53146) twice with 1mL BufferD, 80μL of 50% bead slurry were added to 300μL lysate and incubated at 4 degrees C for 1 hour with rotation. Supernatant was retained as cytoplasmic fraction, and beads were washed three times with 1mL BufferD, eluted with 40μL Laemmli Sample Buffer 4x (containing 10% beta- mercaptoethanol) at 37°C for 30 minutes, separated by 10% SDS-PAGE, transferred to 0.45um nitrocellulose, and probed with anti-DAT antibody (Millipore-Sigma, AB5802). Fluorescent images were analyzed using Fiji/ImageJ (NIH) to measure image density of DAT bands. Values were normalized to total protein per lane. Beta-tubulin (Encor, MCA 1B12) was probed to demonstrate membrane fraction isolation during biotinylation.

Immunocytochemistry: Un-stimulated and LPS-stimulated cells were washed 3 times with 1x external solution and then incubated with CTxB conjugated to AlexaFluor 555 (1:500, Invitrogen, C-34776) +/- LPS in 1x external solution with rotation for 30 minutes at 4°C to limit internalization. Following this, cells were fixed in 4% PFA for 30 minutes at room temperature, and then blocked/permeabilized with 5% normal goat serum, 0.5% Triton-X in PBS for 1 hour at 37°C and 5% CO_2. After blocking, cells were stained with anti-DAT antibody (1:1000, MAB369, Millipore, Berlington MA) in 0.1% Triton-X, 5% normal goat serum in 1xPBS and incubated overnight at 4°C with rotation. Cells were then washed 3 times with PBS for 30 minutes, stained with anti-rat AlexaFluor-647 (1:1000, Invitrogen, A-21247) secondary antibody in 0.1% Triton-X, 5% normal goat serum in 1x PBS for 1 hour at room temperature, then washed again as described above. For DAT/IBA1, NET/IBA1, or SERT/IBA1 immunofluorescent labeling, this process was repeated without the initial CTxB-555 incubation. Primary antibodies, in addition to MAB369, were rabbit anti-IBA1 (1:1000, Encor, RPCA-IBA1), mouse anti-NET (1:100), and mouse anti-SERT (1:2500). Secondary antibodies included anti-rat AlexaFluor-647 (1:1000, Invitrogen, A21247), anti-mouse

AlexaFluor-647 (1:1000, Invitrogen, A-21235), and anti-rabbit AlexaFluor-568 (1:1000, Invitrogen, A-11011). Cells were stored in foil at 4°C until ready for imaging. To determine specificity, one dish per experiment was stained only with secondary antibody. Supplemental Table 2 outlines the source and concentrations of antibodies used in this study.

Fixed Cell Confocal Imaging and analysis for DAT/NET/SERT expression and co-localization: Immuno-stained samples were imaged using a Nikon A1 laser-scanning confocal microscope (Nikon Instruments, Melville NY) using a x60 1.4NA oil immersion objective. Excitation of fluorophores was accomplished using argon lasers of wavelength 561nm and 640nm. Emission was detected at 595/50nm and 700/75nm, respectively. To prevent bleed through, signals were acquired in sequential series. Z-stack images were taken from the basal membrane plane through the top of the cell. For co-expression of IBA1 with DAT, NET, or SERT, maximum intensity projections were generated in Nikon NIS Elements Analysis software (Nikon Instruments, Melville, NY).

To calculate co-localization, for each z-stack, only the z-planes of stacked membrane were taken for analysis. Regions of interest were manually drawn around cells and co-localization between the CTxB555 and DAT signals, as measured by Pearson's correlation coefficient, was taken as the average for each region of interest through the selected z-planes using Nikon Element NIS Analysis software. Individual Pearson's correlation coefficient values were averaged to establish the mean Pearson's correlation coefficient for the image. Image processing for representative images (e.g., rolling-ball background subtraction or denoising) was performed using Nikon Elements NIS analysis software (Nikon Instruments, Melville, NY).

Fixed-cell Total Internal Reflective Fluorescence Microscopy (TIRF-M): CFP-DAT expressing CHO cells were fixed and immuno-labelled for DAT (1:1000, MAB369, Millipore) as above. Macrophages were labelled with CTxB-555 and with anti-DAT antibody (1:1000, MAB369, Millipore), mouse anti-NET (1:100), or mouse anti-SERT (1:2500) as above. The exception is that secondary labeling was accomplished using anti-rat DyLight 405 (1:5000, Novus, NBPI-72983) or anti-mouse DyLight 405 (1:1000, BioLegend, Poly-24091). Fixed cells were then imaged on a Nikon Eclipse TE2000 inverted microscope using a 60x 1.49NA oil-immersion TIRF objective and 405nm and 560nm solid lasers using a CoolSNAP HQ2 CCD camera. The lasers were each aligned manually. Cells were first imaged in brightfield. The TIRF plane was identified using the CFP laser line for CFP-DAT CHO cells and the RFP laser line for macrophages. The TIRF laser incident angle was adjusted manually for each experiment. For each field of view, images in the CFP channel and RFP channel were acquired sequentially, and this process was repeated for multiple fields of view. Image processing for representative images (e.g. background correction and denoising) was performed using Nikon Elements NIS Analysis software.

STED Microscopy: Macrophages were prepared for fixed cell imaging as described above. STED imaging was performed on an Abberior Instruments Expert Line microscope (Abberior Instruments, Germany) mounted on Olympus IX83 microscope operated by Imspector software (version 16.3.11961-w2026; Abberior Instruments, Germany). Alexa 647 was imaged with a 640-nm pulsed excitation laser and a pulsed 775-nm STED laser (5 % of 1.25 W power). Alexa 555 was imaged with a 561-nm pulsed excitation laser and the 775-nm STED laser (40 % of the power). An oil- immersion objective lens (UPlanSApo 100xO NA1.4, Olympus, Japan) was used for imaging. The xy images were recorded with a pixel size of 20 nm in the x and y directions and a pixel dwell time of 10 µs. The xz images were acquired with 100 % of 3D-STED, a pixel size of 86 nm in the z direction, and a y step size of 1 µm.

Analysis of previously published single-cell RNA sequencing data: A unique molecular identifier count matrix was obtained from Li et al. 2020 via the NCBI's Gene Expression Omnibus

by searching for terms "human" and "gut-resident macrophage" or "human" and "intestinal macrophage". Details regarding the acquisition of the original data set can be found in [4].The resulting count matrix was imported into R using the Read10x function (part of the Seurat package)[5]. Quality control, normalization and scaling, principal component identification, clustering, and dimensionality reduction were all carried out using the Seurat pipeline, with default settings as previously described, to generate a t-SNE plot with 13 distinct clusters. Additionally, expression of PTRPP (CD45) as a feature plot to identify leukocytes, and expressions of SLC6A3, AIF1, FGCR2A, CD80, Siglec, and IL-10 as violin plots to annotate cluster 7 were all accomplished via the Seurat pipeline.

3H-Dopamine (3H-DA) Uptake: Human macrophages were plated in a 24 multi-well plate and cultured for 6 days. The experiments were performed in either external solution containing NET and SERT blockers (1µM desipramine and 1µM fluoxetine), or external solution containing NET, SERT, and DAT blockers (10µM desipramine, 1µM fluoxetine, and 5µM nomifensine). Media was aspirated and replaced with 200µL uptake buffer with either 10µM desipramine and 1µM fluoxetine or 10µM desipramine and 1µM fluoxetine and 5µM nomifensine, and 5µL of 0, 0.3, 3.0, 30, 100, or 300µM cold DA, then 50µL of 3H-DA was added to each well. The 300µM cold DA condition measures non-specific DA uptake. After incubation, the uptake solution was removed; the cells were washed, then lysed with SDS for 1 hour while rocking at room temperature. The lysate was collected, and 3H-DA content was measured.

IDT307 Uptake: Human macrophages were cultured on round coverslips for 4-6 days. On the day of experiment, the coverslips were placed into an imaging chamber (Warner Instruments, RC-26G) and washed with 1x external solution. Perfusion throughout the experiments was accomplished using a Warner VC-8 automated perfusion system (Warner Instruments, RC-26G) with the flow rate adjusted to approximately 2-3ml/min. All experiments were performed on a Nikon FN-1 upright microscope (Nikon Instruments, Melville, NY) using a Nikon 40x 0.8NA NIR APO objective. A phase contrast image was taken prior to and following each experiment to monitor cell health. Throughout the experiment, a SpectraX Light Engine (Lumencor, Beaverton OR) was used to emit a 480nm excitation laser at 5% power passed through a quadruple bandpass filter (Chroma Technology Corp., Bellows Falls VT). Images were acquired every 1 second with 100ms exposure with an Andor Zyla 4.2 PLUS camera (Andor Technology, Belfast, UK).

For Figure 3, baseline and IDT perfusion solutions either contained (1) no antagonists, (2) blockade against NET/SERT (desipramine 10µM, fluoxetine 1µM) to isolate DAT; (3) blockade against DAT/SERT (nomifensine 5µM, fluoxetine 1µM) to isolate NET; and complete antagonist cocktail to eliminate specific blinding. For remaining experiments, coverslips were first perfused for 1 minute with external solution, followed by external solution containing specific NET and SERT blockers (10µM desipramine and 1µM fluoxetine), or external solution containing NET, SERT, and DAT blockers (10µM desipramine, 1µM fluoxetine, and 5µM nomifensine) to establish baseline fluorescence values. Once baseline was established, coverslips were perfused for 4 minutes with 5µM IDT307 (Sigma Aldrich, St. Louis, Mo), a fluorescent substrate for neurotransmitter transporters. In parallel experiments, the DAT-mediated substrate (IDT307) uptake was measured in the unstimulated and LPS-stimulated human macrophages. For PMA-induced change in uptake, unstimulated macrophages were treated with 1µM PMA in 1x external for 15 minutes prior to perfusion. Changes in intracellular fluorescence levels (indication of DAT-mediated substrate uptake) were measured for each cell using Nikon NIS-Elements software with background subtraction correction. Each cell served at its own control, where the fluorescence signals were normalized to the cell's baseline fluorescence signal (average fluorescence value across first 60 seconds). DAT-dependent (nomifensine-sensitive) uptake was measured by subtracting the normalized fluorescence profile of the nomifensine blockade condition from the

Desipramine/Fluoxetine condition. Area under curve for each condition was calculated using Prism 7.0 software (GraphPad) to measure cumulative uptake throughout the perfusion period.

JHC1-064 Binding: JHC1-064 was a generous gift from Dr. Amy Newman at the National Institute for Drug Abuse. For Figure 2, macrophages were pre-treated with (1) no antagonists; (2) blockade against NET/SERT (desipramine 10μM, fluoxetine 1μM) to isolate DAT; (3) blockade against DAT/SERT (nomifensine 5μM, fluoxetine 1μM) to isolate NET; and triple antagonist cocktail to eliminate specific blinding. For Supplemental Figure 2, CFP-DAT CHO cells (positive control group) were pre-treated with either vehicle or nomifensine (5μM). For Figure 7, un-stimulated or LPS-stimulated human macrophages were incubated in the external solution supplemented with 1μM desipramine and 1μM fluoxetine for 5 minutes. The fluorescence baseline was taken for 1 minute. JHC1-064, a fluorescent analog of cocaine, which binds to DAT [6], was then added to a final concentration of 50nM. Cells were imaged on a Nikon A1 laser-scanning confocal microscope with a 60x 1.4NA Nikon Plan-Apo objective, and Nikon "perfect focus" were used for all imaging. JHC1-064 was excited using a solid argon 568nm laser. All imaging was done at 37°C. Images were analyzed by measuring the fluorescence signal in the regions of interest (ROI) manually drawn around cells. Changes in the fluorescence signal at the surface membrane after drug application or vehicle were measured and normalized to each region's baseline fluorescence value to determine fold change in JHC1-064 binding to DAT. JHC1-064 rate of binding calculated as the average slope beginning at 60 seconds, when JHC1-064 perfusion began through the end of recording (GraphPad).

JHC1-064 Total Internal Reflection Fluorescent (TIRF) Microscopy: For Figure 2 and Supplemental Figure 2, YFP-DAT cells (positive control group) or human macrophages were treated as above. Following binding, observed via confocal microscopy, dishes were transferred to an inverted Nikon Eclipse TE200 microscope equipped with solid 405, 515, and 560nm lasers and a 60x 1.49NA oil-immersion objective for TIRF-M imaging, as above, using the 560nm (RFP laser line only). Snapshot images of the TIRF plane were taken using the 560nm laser. For Supplemental Figure 6, macrophages were incubated in 40nM JHC1-064 solution for 30 min at room temperature as described previously [7]. The live-cell TIRF imaging was done at 37°C using an objective heater (20/20 Technology Inc). The JHC1-064 binding was confirmed via live cell imaging; dishes were then washed to remove unbound JHC1-064, then placed back on the TIRF microscopy system (Richardson et al. 2016). Baseline fluorescence was recorded for 30s before application of either vehicle or 1μM phorbol 12-myristate 13- acetate (PMA) (Sigma Aldrich, Cat# P8139) and imaged every 10s for 5 minutes at 50% laser power. The data were analyzed as we have described previously(Richardson et al. 2016). Regions of interest were manually drawn around cells and fluorescence was measured throughout the experiment in each ROI using Nikon Elements NIS analysis software. The change in fluorescence was calculated as (f-fbaseline)/fbaseline x 100%.

Whole-cell Electrophysiology Recordings: Cells/macrophages were visualized using an Andor Zyla 4.2 PLUS camera and a 40x Nikon 0.8NA Plan-APO objective for the duration of the experiment on a Nikon FN-1 upright microscope in the presence of NET/SERT blockade (10μM desipramine, 1μM fluoxetine). Whole cell voltage-clamp recordings were taken using an Axopatch 200B amplifier and Digidata 1440A low-noise data acquisition system. All parameters were monitored using Axon Clampex software (Molecular Devices, Sunnyvale CA). Low noise borosilicate glass pipettes (outer diameter: 1.0mm; inner diameter: 0.78mm; Sutter Instruments Cat. BF100-78-10) were pulled using a laser-based pipette puller (Model P-2000, Sutter Instruments Co, Novato CA) with a tip resistance of 2-5MΩ. The data were analyzed using Clamp-fit software (Molecular Devices, Sunnyvale CA). First, cells/macrophages were patched in cell-attached configuration with a membrane resistance of ≥1GΩ, and then the intracellular

compartment was accessed via rapid application of negative pressure, switching to whole-cell configuration. The DAT-mediated inward current was measured in YFP-DAT cells (positive control group), and in un-stimulated and LPS-stimulated macrophages via whole cell voltage-clamp recordings as we have described previously [7]. Briefly, once a stable patch-clamp was established in the whole-cell configuration at a holding voltage of -40mV, a voltage step protocol was run from 0 to -120 mV in 20mV increments to establish a baseline voltage current relationship, I(V). Each I(V) protocol was separated by at least 2-3 min to ensure cell viability. After acquiring baseline, I(V), nomifensine was applied to measure uncoupled DAT-mediated current (baseline current minus current after nomifensine,10μM) [7]. The amphetamine- induced, DAT-mediated current is defined as amphetamine-induced current minus nomifensine-induced current [7].

Amperometry: Single cell simultaneous patch-clamp and amperometry experiments were performed as described previously [8,9]. The amperometric carbon-fiber electrode (ProCFE, Dagan Corp.) was connected to an Axopatch 200B amplifier (Molecular Devices, Sunnyvale CA). In the presence of NET/SERT blockade (10μM desipramine, 1μM fluoxetine), macrophages were patch-clamped in cell-attached configuration, first with minimum 1GΩ membrane resistance, and then switched to whole-cell configuration by rapid application of negative pressure and dialyzed with dopamine via the patch electrode. The amperometry electrode was placed near the cell and held at +700mV. Oxidative currents were generated by a voltage-step protocol starting at 100mV and decreasing to 60mV in 20mV increments and recorded via the amperometry electrode. Currents were recorded at baseline (5 minutes after whole-cell configuration was achieved), 3 min post amphetamine addition, and 3 min post nomifensine addition. Recordings were low-pass filtered at 60Hz for subsequent analysis. Efflux was reported as the averaged difference between the last 100ms of the voltage step and the 100ms prior to the voltage step. Baseline and the amphetamine-induced, DAT-dependent dopamine efflux were calculated by subtracting the efflux after nomifensine treatment.

MitoSOX™ Imaging: Human monocyte-derived macrophages from healthy donors were cultured as described above on 12 mm #1.5 round coverslips. Complete, fresh MoAM was supplied to the macrophages on day 3 and experiments were conducted on days 6-7. Macrophages were given fresh, nomifensine-supplemented, LPS-supplemented, or nomifensine- and LPS-supplemented complete MoAM and incubated at 37°C and 5% CO2 for 24 hours before beginning experiments.

Thirty minutes prior to live-cell imaging, 500μL of conditioned media was collected from each 1mL well for AlphaLISA assay and macrophages were loaded with MitoSOX™ Red (Sigma Aldrich, St. Louis, Mo) mitochondrial superoxide indicator at a concentration of 1μM in complete, fresh MoAM at 37°C. Coverslips were then placed in an imaging chamber (Warner Instruments) and briefly perfused with 1x external solution at room temperature prior to imaging. Experiments were carried out on the Nikon FN-1 microscope used for IDT307 uptake with the same objective lens. A 555nm excitation laser passed through a quadruple bandpass filter at 25% power during the experiment. Images were captured with an Andor Zyla 4.2 PLUS camera and analyzed using Nikon NIS-Elements software. Objects were identified based on a fluorescence intensity threshold and converted to ROIs. Mean fluorescence intensity of each ROI was taken as a measure of mitochondrial oxidation for each individual macrophage.

Fixed-cell phagocytosis assay: We adapted a recently published protocol for fixed-cell analysis of phagocytosis in vitro [10]. Cultured macrophages were treated with either (1) vehicle (complete media), (2) LPS (10ng/ml), (3), LPS + nomifensine (10μM), (4) LPS + nomifensine + dopamine (1μM), or (5) LPS + nomifensine + dopamine + Sulpiride (5μM) + SCH23390 (5μM). All conditions also contained .007g/ml of ascorbic acid to minimize dopamine oxidation (pH balanced to approximately 7.4 with NaOH) and desipramine (10μM). 1-2 wells/condition also received 0.1μg/ml of fluorescent latex beads. Macrophages were incubated at 37ºC for 2hr. Following

incubation, 400μl of conditioned media from wells without phagocytic beads was collected for AlphaLisa assay (see below). Macrophages were washed 3x with PBS and then fixed in 4% PFA at room temperature for 20 minutes. Following fixation, macrophages were blocked/permeabilized using 0.3% Triton-X and 5% normal goat serum in PBS for at least 1hr at 37ºC. Macrophages were then incubated with anti-IBA1 from rabbit (1:1000, Encor) overnight at 4ºC on a rocker. Following primary incubation, macrophages were washed 3x with PBS with moderate rocking at room temperature followed by a 1hr incubation with anti-rabbit AlexaFluor-488 (1:1000, Invitrogen) at room temperature. After 3x 20min PBS washes, coverslips were mounted and allowed to dry overnight.

Macrophages were imaged using a Nikon A1 laser scanning confocal microscope and a 40x oil-immersion 1.3NA Plan-Apo objective. The IBA1 and fluorescent beads were excited by 488nm and 561nm solid argon lasers, respectively, and detected at 525nm and 595nm, respectively. To prevent bleed through, lasers were fired in a sequential series. Multiple z-stacks taken through the depth of the cells were acquired for each coverslip. Images were then imported into Fiji/ImageJ for blinded analysis and the channels were separated. The 488nm channel (IBA1) was collapsed into a maximum intensity projection and regions of interest were manually drawn around each cell. The integrated fluorescence density for each region of interest from the 560nm channel (beads) was extracted and pasted into an excel sheet. This process was repeated for each image, from each coverslip, for each experiment. The vehicle, LPS, and LPS+Nom conditions from the first three experiments were analyzed to generate the data in Figure 5. The later experiments were analyzed to generate the data in Figure 9. The data table was uploaded into a publicly available pipeline for statistical analysis. The graphical interface generates frequency histograms and cumulative probability distributions for the data set and runs sequential Kolmogorov-Smirnov tests. These are the statistics reported in the results, figures, and figure legends. The complete code and link to the graphical user interface are available at: Simultaneously, the data were uploaded into R to generate cumulative frequency distributions aesthetically tailored to the color scheme of the rest of the figures, and the data were uploaded into GraphPad to generate plots comparing median fluorescence.

AlphaLISA assay: Monocyte-derived macrophages were treated as described above and supernatants were collected prior to microscopy assays. Supernatants were shipped on dry ice to the Gaskill Lab at Drexel University, where they were analyzed for IL-6, TNF-α, CCL2 using AlphaLISAs performed according to the manufacturer's protocol (PerkinElmer). Briefly, supernatants were thawed and aliquoted in duplicate into 1/2 area, opaque white 96 well plates (#6002290, Perkin Elmer) at 5 μL per well. Following addition of sample, master mix containing acceptor beads and antibody is added to each well, and plates are incubated in the dark at room temp on a high-speed shaker for 60 minutes. After 60 minutes, donor beads are added, and plate is again incubated at room temperature in the dark on a high-speed shaker for 30 minutes. After second incubation, plates are read on an Enspire Multimode Plate reader using the AlphaLISA setting. The lower limits of detection for AlphaLISAs were IL-6, 1.3 pg/ mL; TNF-α, 2.2 pg/mL; CCL2, 3.8 pg/ml. The limit of quantification for these assays was 10 pg/mL for TNF-a and IL-6, and 30 pg/mL for CCL2.

IL-1β ELISA: Human macrophages were cultured at a density of 100,000 cells/well and treated with either vehicle, LPS, nomifensine, or LPS+nomifensine as described above. Following treatment, cells were lysed in the presence of a protease inhibitor cocktail. Cell lysate was collected, then analyzed for IL-1β using an IL-1β ELISA (BioLegend, 437004, San Diego CA) according to the manufacturer protocol.

Statistical Analysis

Data analysis was performed via Prism 7 (GraphPad Software Inc.) or, for phagocytosis analysis, in R/an R-encoded interface. Sample sizes were not determined by sample size calculations but represent sample sizes similar to those generally used in the field for the respective types of experiments. Normality and lognormality was assessed using Kolmogrov-Smirnov test for Figures 2 and 3, and either Shapiro-Wilkes or D'Agnostico-Pearson test for the remaining figures. N was reported as number of cells when validating pharmacology and basic transporter function. Also, for phagocytosis, n was reported as number of cells to take a whole-population approach as previously described. Otherwise, n represent independent experiments. Bar graphs are depicted as mean ± SEM with individual points. One-way, two-way, or repeated measures ANOVA (with multiple comparison tests as indicated for each experiment) or Kruskal-Wallis test with Dunn's test for multiple comparisons were used for the statistical comparison of more than two means of data. Unpaired t-tests or Mann-Whitney tests were used to compare two means of data. Comparisons in variance were done by computing the F-statistic in Prism. For significantly different variances ($p<0.05$) either Brown-Forsythe or Welch's corrections were applied for ANOVA or t-tests, respectively. Kolmogorov-Smirnov tests were used to compare non-normal cumulative frequency distributions. Significance at $p \leq 0.05$ was considered statistically significant and are reported to the first significant figure. Details on statistical tests and n can be found in each figure legend.

Table S1: Inhibitors/Fluorescent Substrates

Reagent	Supplier	Cat. No.	Final Concentration	Specificity	Reference
Nomifensine	Sigma Aldrich	N1530	5-20μM	IC50=7.2nM	[11]
GBR	Sigma Aldrich	G9659	10μM	Ki=21.5nM	Sigma
Desipramine	Sigma Aldrich	D3900	1μM	Ki=4.1nM	Sigma
Fluoxetine	Tocris	0927	1μM	Ki=0.9nM	Tocris
CLI-095	Invivogen	tlrl-cl95	3μM	IC50=1-35nM	Li M. et al 2006
IDT307	Sigma Aldrich	SML0756	5μM	Km=30μM	[12]
JHC1-064	Dr. Amy Newman (NIDA)	-	50nM	Ki=18nM	Cha et al. 2005
anti-CD14	Biolegend	367102	10μg/ml	-	Kurt-Jones E.A. et al. 2000
Iaxo-102	AdipoGen	IAX-600-002-M001	5μM	-	[13]
Sulpiride	Sigma Aldrich	S8010	5μM	Ki≈10nM	
SCH23390	Sigma Aldrich	D054	5μM	Ki=0.12nM	[14]
Avidin Biotin Blocking system	Biolegend	927301	-	-	-

Table S2: Primary Antibodies/Fluorescent Dyes				
**Antibody**	**Supplier**	**Cat. No./RRID**	**Dilution**	**Application**
Rat anti-DAT	Millipore	Mab369/ AB_2190413	1:500	IF, WB
Mouse anti-TH	Millipore	T1299/ AB_477560	1:500	IF
Rabbit anti-IBA1	WAKO	LKF6437	1:500	IF
CTxB-Alexa Fluor 555	Thermo Fisher	C34776	1:500	IF
anti-beta tubulin	Encor	MCA 1B12	1:40,000	WB
Rabbit anti-IBA1	Encor	RPCA-IBA1	1:1000, 1:800	IF
Mouse anti-NET	MabTechnologies	NET17-1	1:100; 1:100; 1:1000	IF, FC, WB
Mouse anti-SERT	MabTechnologies	ST51-2	1:2500; 1:100 1:5000	IF, FC, WB
Chicken anti-MAP2	BioLegend	Poly28225	1:800	IF
Anti-TNF PE	BioLegend	502908	1:100	FC
Anti-IL6 PE-Dazzle594	BioLegend	501121	1:100	FC
Anti-CCL2 APC	BioLegend	505909	1:100	FC
Anti-IL1β Pacific Blue	BioLegend	511709	1:100	FC
Fluorescent Latex Beads	Sigma Aldrich	L3280	1µg/ml	IF
MitoSox Red™	Thermo Fisher	M36009	1µM	Live imaging
False Fluorescent Neurotransmitter 200	Tocris	5911	20µM	Live Imaging
Anti-Rat Dylight 405	Novus	NBPI-72983	1:5000	IF
Anti-Rat AlexaFluor 568	Invitrogen	A11-077	1:1000	IF
Anti-Rat AlexaFluor 647	Invitrogen	A-21247	1:1000	IF
Anti-Rat Biotin	Vector	BA9401	1:200	IF
Anti-Mouse DyLight 405	BioLegend	Poly24091	1:1000	IF
Anti-Mouse AlexaFluor 647	Invitrogen	A21235`	1:1000	IF
Anti-Rabbit AlexaFluor 568	Invitrogen	A11011	1:800-1:1000	IF
Anti-Chicken AlexaFluor 488+	Invitrogen	A32931	1:800	IF
Streptavidin AlexaFluor 647	Invitrogen	S32357	1:200	IF

Table S3: Equipment used for microscopy and electrophysiology experiments

Equipment	Experiment used	Source
Nikon A1 laser-scanning confocal microscope	Live or Fixed cell confocal Imaging	Nikon Instruments, Melville NY
60x 1.49 NA Nikon Plan-Apo objective	Live or Fixed cell confocal Imaging	Nikon Instruments, Melville NY
NIS Elements analysis software	Image analysis	Nikon Instruments, Melville NY
480nm excitation laser equipped with a quadruple bandpass filer	Imaging	Chroma Technology Corp., Bellows Falls VT
FN-1 upright microscope	IDT307 Uptake	Warner Instruments, RC-26G
SpectraX Light Engine	IDT307 Uptake	Lumencor, Beaverton OR
Andor Zyla 4.2 PLUS camera	Imaging	Andor Technology, Belfast, UK
Nikon Eclipse TE 2000-U Inverted Microscope	TIRF microscopy	Nikon Instruments, Melville NY
40x 1.49 NA objective	TIRF microscopy	Nikon Instruments, Melville NY
40x Nikon 0.8NA Plan-APO objective	Electrophysiology and amperometry	Nikon Instruments, Melville NY
Amperometric carbon-fiber electrode	Amperometry	ProCFE, Dagan Corp
Axopatch 200B amplifier	Electrophysiology and amperometry	Molecular Devices, Sunnyvale CA
Digidata 1440A low-noise data acquisition system	Electrophysiology and amperometry	Molecular Devices, Sunnyvale CA
Axon Clampex software	Electrophysiology and amperometry	Molecular Devices, Sunnyvale CA
Clamp-fit software	Electrophysiology and amperometry	Sutter Instruments Co, Novato CA
Laser-based pipette puller, Model P-2000	Electrophysiology and amperometry	Sutter Instruments Co, Novato CA
Imaging chamber	microscopy and electrophysiology	Warner Instruments, RC-26G
Warner VC-8 automated perfusion system	Perfusion for drug application, solution exchange	Warner Instruments, RC-26G

References

1 Dagur, P. K. & McCoy, J. P., Jr. Collection, Storage, and Preparation of Human Blood Cells. *Curr Protoc Cytom* **73**, 5.1.1-16, doi:10.1002/0471142956.cy0501s73 (2015).

2 Gopinath, A. *et al.* A novel approach to study markers of dopamine signaling in peripheral immune cells. *J Immunol Methods* **476**, 112686, doi:10.1016/j.jim.2019.112686 (2020).

3 Ii, M. *et al.* A novel cyclohexene derivative, ethyl (6R)-6-[N-(2-Chloro-4-fluorophenyl)sulfamoyl]cyclohex-1-ene-1-carboxylate (TAK-242), selectively inhibits toll-like receptor 4-mediated cytokine production through suppression of intracellular signaling. *Mol Pharmacol* **69**, 1288-1295, doi:10.1124/mol.105.019695 (2006).

4 Li, J. *et al.* Genomic and transcriptomic profiling of carcinogenesis in patients with familial adenomatous polyposis. **69**, 1283-1293 (2020).

5 Satija, R., Farrell, J. A., Gennert, D., Schier, A. F. & Regev, A. Spatial reconstruction of single-cell gene expression data. **33**, 495-502 (2015).

6 Cha, J. H. *et al.* Rhodamine-labeled 2beta-carbomethoxy-3beta-(3,4-dichlorophenyl)tropane analogues as high-affinity fluorescent probes for the dopamine transporter. *J Med Chem* **48**, 7513-7516, doi:10.1021/jm050431y (2005).

7 Richardson, B. D. *et al.* Membrane potential shapes regulation of dopamine transporter trafficking at the plasma membrane. *Nat Commun* **7**, 10423, doi:10.1038/ncomms10423 (2016).

8 Khoshbouei, H., Wang, H., Lechleiter, J. D., Javitch, J. A. & Galli, A. Amphetamine-induced Dopamine Efflux: A VOLTAGE-SENSITIVE AND INTRACELLULAR Na+-DEPENDENT MECHANISM. *Journal of Biological Chemistry* **278**, 12070-12077, doi:10.1074/jbc.M212815200 (2003).

9 Sambo, D. O. *et al.* The sigma-1 receptor modulates methamphetamine dysregulation of dopamine neurotransmission. *Nat Commun* **8**, 2228, doi:10.1038/s41467-017-02087-x (2017).

10 Caponegro, M., Thompson, K., Tayyab, M. & Tsirka, S. A Rigorous Quantitative Approach to Analyzing Phagocytosis Assays. **10** (2020).

11 Rothman, R. B. *et al.* Identification of a GBR12935 homolog, LR1111, which is over 4,000-fold selective for the dopamine transporter, relative to serotonin and norepinephrine transporters. *Synapse* **14**, 34-39, doi:doi:10.1002/syn.890140106 (1993).

12 Blakely, R. D., Mason, J. N., Tomlinson, I. D. & Rosenthal, S. J. (Google Patents, 2011).

13 Huggins, C. *et al.* A novel small molecule TLR4 antagonist (IAXO-102) negatively regulates non-hematopoietic toll like receptor 4 signalling and inhibits aortic aneurysms development. **242**, 563-570 (2015).

14 Faedda, G., Kula, N. S. & Baldessarini, R. J. Pharmacology of binding of 3H-SCH-23390 to D-1 dopaminergic receptor sites in rat striatal tissue. **38**, 473-480 (1989).

Supplemental Questionnaire 1: Example LifeSouth criterion for healthy human blood donor

#	YES	NO	
A.	●	○	Can you read, speak, and understand English?
B.	●	○	Is your gender identity the same as your sex at birth?
C.	●	○	Is your information above correct? ○ IBBIS correction made:
			**Donor History Questionnaire**
1.	●	○	Are you feeling healthy and well today?
2.	○	●	Are you currently taking an antibiotic?
3.	○	●	Are you currently taking any other medication for an infection?
4.	○	●	Have you taken any medications on the Medication Deferral List in the time frames indicated? *(Review the Medication Deferral List.)*
5.	●	○	Have you read the Educational Materials today?
6.	○	●	In the past **48 hours**, have you taken aspirin or anything that has aspirin in it?
			In the past 8 weeks, have you
7.	○	●	Donated blood, platelets, or plasma?
8.	○	●	Had any vaccinations or other shots?
9.	○	●	Had contact with someone who was vaccinated for smallpox in the past 8 weeks?
10.	○	●	In the past **16 weeks,** have you donated a double unit of red cells using an apheresis machine?
			In the past 12 months, have you
11.	○	●	Had a blood transfusion?
12.	○	●	Had a transplant such as organ, tissue, or bone marrow?
13.	○	●	Had a graft such as bone or skin?
14.	○	●	Come into contact with someone else's blood?
15.	○	●	Had an accidental needle stick?
16.	○	●	Had sexual contact with anyone who has HIV/AIDS or has had a positive test for the HIV/AIDS virus?
17.	○	●	Had sexual contact with a prostitute or anyone else who takes money or drugs or other payment for sex?
18.	○	●	Had sexual contact with anyone who has ever used needles to take drugs, steroids, or anything not prescribed by their doctor?
19.	○	●	○ *FEMALE* **Male donors**: Had sexual contact with another male?
20.	○	○	● *MALE* **Female donors**: Had sexual contact with a male who had sexual contact with another male in the past 12 months?

#	YES	NO	***In the past 12 months, have you***
21.	○	●	Had sexual contact with a person who has hepatitis?
22.	○	●	Lived with a person who has hepatitis?
23.	○	●	Had a tattoo?
24.	○	●	Had ear or body piercing?
25.	○	●	Had or been treated for syphilis or gonorrhea?
26.	○	●	Been in juvenile detention, lockup, jail, or prison for more than 72 hours?
27.	○	●	In the past **three years**, have you been outside the United States or Canada?
			From 1980 through 1996,
28.	○	●	Did you spend time that adds up to three months or more in the United Kingdom? *(Review list of countries in the UK.)*
29.	○	●	Were you a member of the U.S. military, a civilian military employee, or a dependent of a member of the U.S. military?
			From 1980 to the present, did you
30.	○	●	Spend time that adds up to five years or more in Europe? *(Review list of countries in Europe.)*
31.	○	●	Receive a blood transfusion in the United Kingdom or France? *(Review country lists.)*
			Have you EVER
32.	○	○	● *MALE* **Female donors**: Been pregnant or are you pregnant now?
33.	○	●	Had a positive test for the HIV/AIDS virus?
34.	○	●	Used needles to take drugs, steroids, or anything not prescribed by your doctor?
35.	○	●	Received money, drugs, or other payment for sex?
36.	○	●	Had malaria?
37.	○	●	Had Chagas' disease?
38.	○	●	Had babesiosis?
39.	○	●	Received a dura mater (or brain covering) graft or xenotransplantation product?
40.	○	●	Had any type of cancer, including leukemia?
41.	○	●	Had any problems with your heart or lungs?
42.	○	●	Had a bleeding condition or a blood disease?
43.	○	●	Have any of your relatives had Creutzfeldt-Jakob disease?
44.	○	●	Do you have any reactions or issues with the blood donation process that we should know about?
